# Newly discovered Late Triassic Baqing eclogite in central Tibet indicates an anticlockwise West–East Qiangtang collision

**DOI:** 10.1038/s41598-018-19342-w

**Published:** 2018-01-17

**Authors:** Yu-Xiu Zhang, Xin Jin, Kai-Jun Zhang, Wei-Dong Sun, Jian-Ming Liu, Xiao-Yao Zhou, Li-Long Yan

**Affiliations:** 1 Asian Tectonics Research Group, College of Earth Science, University of Chinese Academy of Sciences, 19 A Yuquan Road, Beijing, 100049 China; 2 Key Laboratory of Computational Geodynamics, Chinese Academy of Sciences, 19 A Yuquan Road, Beijing, 100049 China; 3 Center of Deep Sea Research, Institute of Oceanology, Chinese Academy of Sciences, Qingdao, 266071 China; 4 CAS Center for Excellence in Tibetan Plateau Earth Sciences, Chinese Academy of Sciences, Guangzhou, 510640 China

## Abstract

The Triassic eclogite-bearing central Qiangtang metamorphic belt (CQMB) in the northern Tibetan Plateau has been debated whether it is a metamorphic core complex underthrust from the Jinsha Paleo-Tethys or an *in-situ* Shuanghu suture. The CQMB is thus a key issue to elucidate the crustal architecture of the northern Tibetan Plateau, the tectonics of the eastern Tethys, and the petrogenesis of Cenozoic high-K magmatism. We here report the newly discovered Baqing eclogite along the eastern extension of the CQMB near the Baqing town, central Tibet. These eclogites are characterized by the garnet + omphacite + rutile + phengite + quartz assemblages. Primary eclogite-facies metamorphic pressure–temperature estimates yield consistent minimum pressure of 25 ± 1 kbar at 730 ± 60 °C. U–Pb dating on zircons that contain inclusions (garnet + omphacite + rutile + phengite) gave eclogite-facies metamorphic ages of 223 Ma. The geochemical continental crustal signature and the presence of Paleozoic cores in the zircons indicate that the Baqing eclogite formed by continental subduction and marks an eastward-younging anticlockwise West–East Qiangtang collision along the Shuanghu suture from the Middle to Late Triassic.

## Introduction

**Figure 1:**
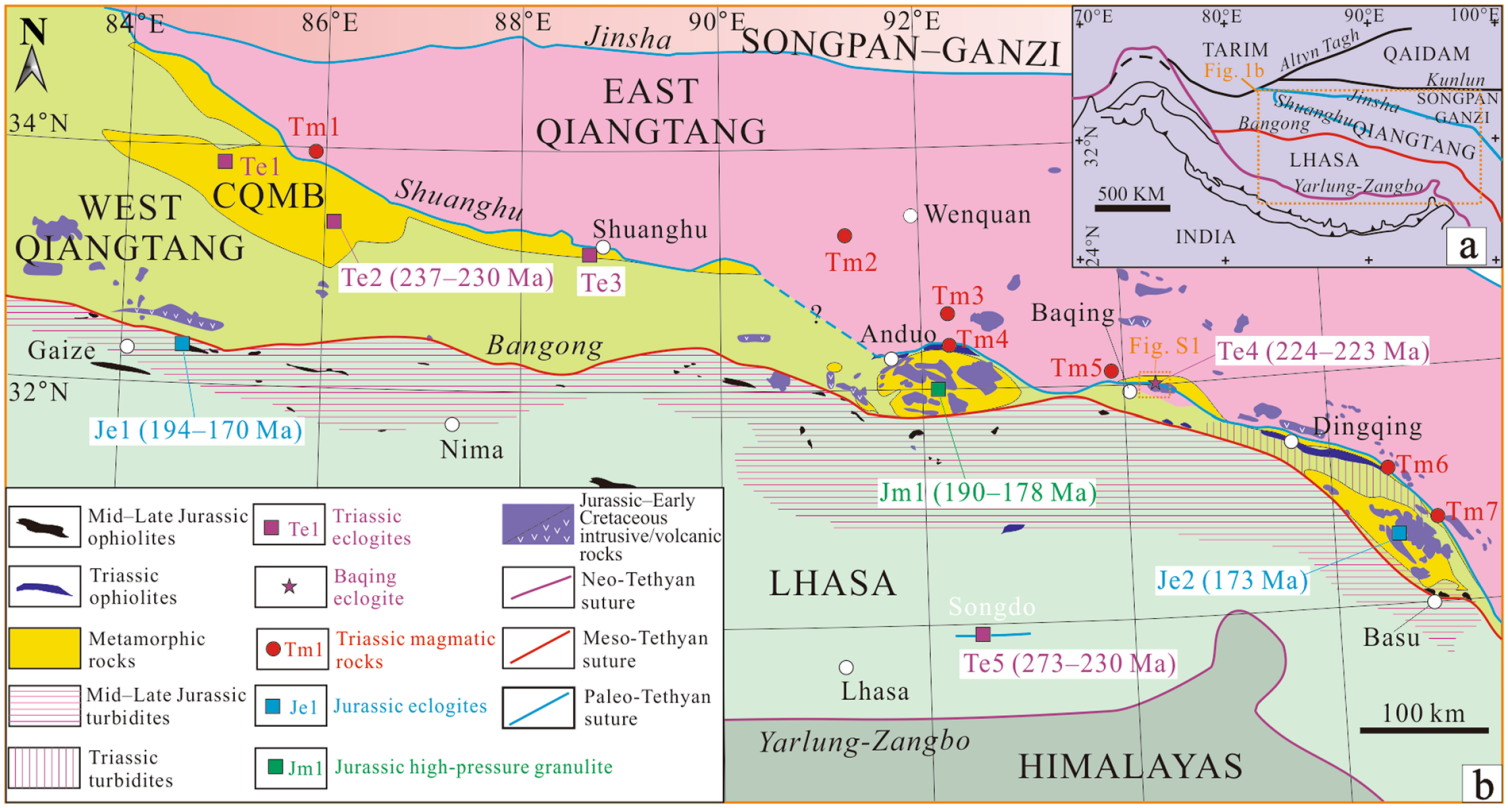
(**a**) Simplified tectonic map of the Tibetan Plateau, western China. (**b**) Main tectono-stratigraphic domains, showing the distribution of eclogites in the interior of the Tibetan Plateau, modified after refs^1,10^. Data sources: Te1–Gangmacuo^9,54^; Te2–Gemuri^4^; Te3–Amugang^10^; Te4–Baqing (this study); Te5–Songduo^55,56^; Je1–Gaize^40^; Je2–Basu^44,45^; Jm1–Anduo^41–43^; Tm1^3,7^; Tm2^57^; Tm3 and Tm4^58^; Tm5, Tm6, and Tm7^48^. This figure is generated by Kai-Jun Zhang and Yu-Xiu Zhang, using CorelDRAW X6 created by the CorelDRAW Team under an open license (http://www.coreldraw.com/cn/product/graphic-design-sofware/).

The >500-km-long and up to 100-km-wide Triassic eclogite- and blueschist-bearing central Qiangtang metamorphic belt (CQMB) in the western–central Qiangtang area, northern Tibetan Plateau^1–5^ (Fig. 1a,b) has been widely debated whether it is a metamorphic core complex southerly underthrust over the oceanic lithosphere at a low angle from the Jinsha Paleo-Tethyan suture zone and then exhumed in the Qiangtang interior^2,3,6,7^, or an *in situ* Shuanghu Paleo-Tethyan suture zone^4,8,9^. The underthrusting model predicts the present-day deep crust–lithosphere mantle of the East Qiangtang subterrane is completely replaced by oceanic crust-lithosphere mantle carrying voluminous early Mesozoic mélange while the *in-situ* model predicts a normally thickened continental crustal structure that resulted from the West–East Qiangtang continental collision. Furthermore, the underthrusting model predicts the enigmatic Cenozoic magmatism in the whole northern Qiangtang area could have been produced by melting of the underthrust mélange whereas the *in-situ* model predicts the magmatism formed by melting of delaminated continental crust/lithosphere in response to the Indo–Asia collision^1–3^. The CQMB thus is essential to elucidate the crustal structure of the northern Tibetan Plateau, the tectonic framework and evolution of the Paleo-Tethyan realm, and the petrogenesis of Cenozoic high-K magmatism^2–4,8,10^. Although the discovery of western CQMB eclogite^4^ provides pivotal research materials, such debate was still existing mainly due to the absence of blueschist and/or eclogite along the extension of the CQMB in the eastern–central Qiangtang area (Fig. 1a,b). In this study, we report for the first time the occurrence, mineralogy, geochemistry and geochronology of eclogite discovered near the Baqing town in the eastern Qiangtang area, central Tibet. Our findings have important implications for the evolution of the Paleo-Tethys and the crustal architecture of the northern Tibetan Plateau.

## Geologic setting and field relations

The Qiangtang terrane in the northern Tibetan Plateau represents Gondwana-derived continental fragments and is bounded to the north by the Jinsha Paleo-Tethyan suture and to the south by the Bangong Meso-Tethyan suture, respectively (Fig. 1a,b). The Qiangtang terrane consists of the West and East subterranes, separated by the CQMB, but its extension in the eastern–central Qiangtang region is ambiguous^5,10^ (Fig. 1a,b). The discovery of Proterozoic gneissic granite and metamorphic volcanic rocks (1048–991 Ma)^11^ indicates that the Qiangtang terrane is underlain by Proterozoic basement. The terrane received passive margin-type stable marine sedimentation through the Paleozoic and developed into large-scale foreland basins during Late Triassic–Jurassic period, characterized by thick accumulation of siliciclastic rocks of recycled-orogen origin intercalated with minor limestone^1,12,13^. The western CQMB eclogite and blueschist occur as blocks in garnet-mica-quartz schist, marble or metampelite, with eclogite-facies mineral assemblage of garnet + omphacite + rutile + quartz consistent with pressure of 20–25 kbar at 410–460 °C^4,9^. Both the eclogite and blueschist show a geochemical affinity with ocean island basalt (OIB) or enriched mid-ocean ridge basalt (E-MORB)^4,8,14^. The eclogite- and blueschist-bearing high-pressure (HP) mélange of the western CQMB, and the Triassic arc-related magmatic rocks exposed in the East Qiangtang subterrane mark a northward subduction of the Shuanghu Paleo-Tethys^3,7,8,15^ (Fig. 1b).

The eastern CQMB is mainly composed of an association of garnet-mica-quartz schist, marble, metapelite and meta-sandstone^10,16^, ~100 km north of the conventionally-called Bangong Meso-Tethyan suture zone (Figs 1b, S1). The newly discovered eclogite bodies (named Baqing eclogite in this study) occur within the eastern CQMB, ~70 km northeast of the Baqing town (Fig. 1b). The eclogite-bearing eastern CQMB is unconformably overlain by Uppermost Triassic–Jurassic sedimentary rocks of coastal sandstone, mudstone with minor limestone^1,8^ (Fig. S1). So far, we have found the Baqing eclogite bodies are exposed in a zone of >10 km long and ~100 m wide in a northwest–southeast extension (Fig. S1). The eclogite pods are generally massive at outcrop scale (Figs S1, S2). They occur as variably retrogressed elongate lens-like boudins, a few centimeters to tens of meters in size, with long axis sub-parallel to the foliation strike of the host schists (Figs S1, S2). These Baqing eclogite boudins juxtapose the host garnet-mica-quartz schist with ductile fault contact (Figs S1, S2a). The Baqing eclogite shows coarse-grained texture with mineral assemblages of garnet, omphacite, rutile, phengite, and minor quartz, epidote and titanite (Fig. S2b; Table S1).

## Results

### Petrography and mineralogy

**Figure 2:**
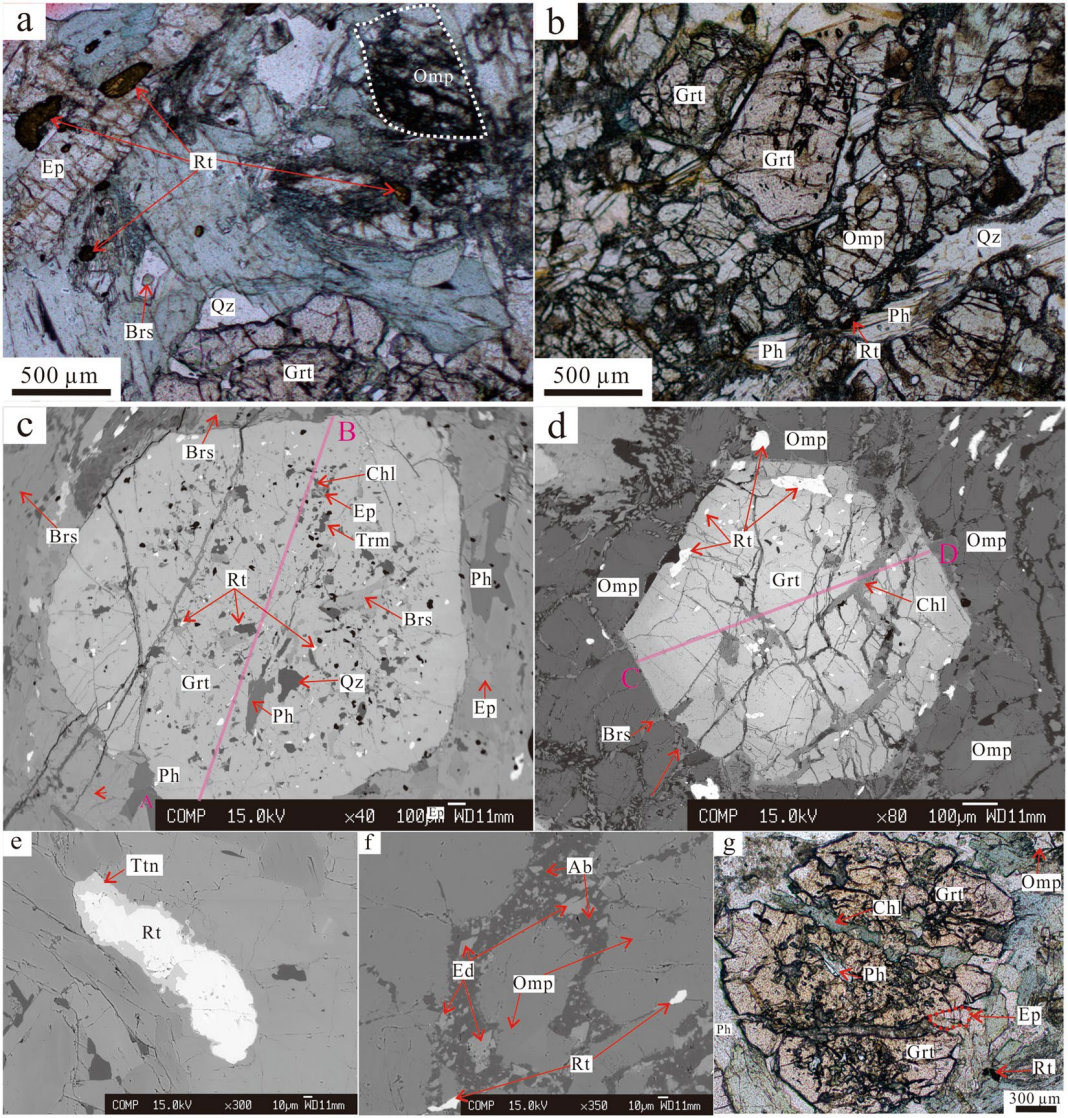
Photomicrographs and backscattered electron (BSE) images. (**a**) Plane-polarized photomicrographs show mineral assemblages of Grt + Omp + Brs + Rt + Ep + Qz (sample YA-7-18-40). (**b**) Plane-polarized photomicrograph shows mineral assemblage of Grt + Omp + Rt + Ph + Qz (sample YA-7-18-47). (**c**) BSE image shows large amount of inclusions in garnet, which are mainly Rt + Brs + Ph + Qz + Ep + Trm + Chl, more abundant in the core than in the rim, and the crack of garnet filled with chlorite (sample YA-7-18-40). Pink line AB shows the garnet compositional profile in Fig. 3a. (**d**) BSE image shows the euhedral garnet contacts directly with omphacite and amphibole (sample YA-7-18-47). Pink line CD shows the garnet compositional profile in Fig. 3b. (**e**) BSE image shows the rim of rutile replaced by titanite (sample YA-7-18-40). (**f**) Mineral assemblage of Grt + Rt + Omp + Ab + Ed (sample YA-7-18-47). Omphacite decomposed into amphibole and albite. (**g**) Plane-polarized photomicrograph shows mineral assemblages of Grt + Ph + Ep + Rt + Omp + Chl, and the crack of garnet is filled with chlorite and epidote (sample YA-7-18-40).

The Baqing eclogite displays granoblastic or weakly foliated texture with symplectites, and consists of garnet (Grt, ~20–35 vol.%), omphacite (Omp, ~10–35 vol.%), phengite (Ph, ~5–10 vol.%), rutile (Rt, <5 vol.%), quartz (Qz, ~5 vol.%), amphibole (Amp, ~5–30 vol.%), chlorite (Chl, <5 vol.%), epidote (Ep, ~5–10 vol.%), albite (Ab, ~5 vol.%), ilmentite (Ilm, <5 vol.%), and titanite (Ttn, ~1–2 vol.%) (Table S1; Fig. 2a–f). The mineral abbreviations are according to ref.^17^. In addition, zircon (Zrn) and apatite (Ap) occur as accessory minerals, and the mineral assemblages are similar to those of western CQMB eclogite^4,9,10^.

**Figure 3:**
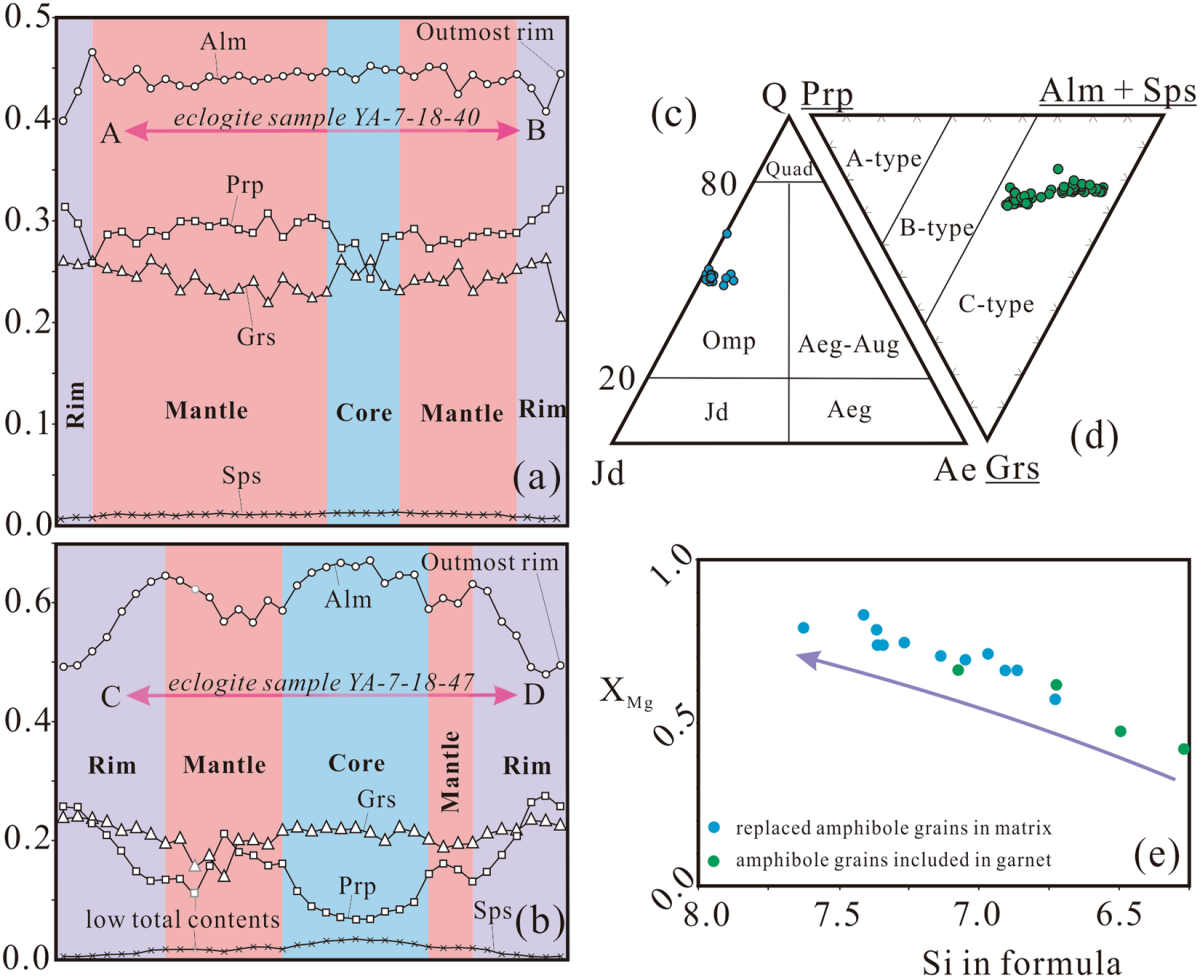
(**a**,**b**) Garnet compositional profiles (see Fig. 2c,d for the locations). (**c**) Omphacite compositions (after ref.^59^). Abbreviations: Quad–Ca–Mg–Fe pyroxene field; Q–quadrilateral pyroxene; Wo–wollastonite; En–enstatite; Fs–ferrosilite; Jd–jadeite; Ae–aegirine. (**d**) Garnet compositions (after ref.^18^). Abbreviations: A-type–inclusions from kimberlites, basalts, or ultramafic rocks layers; B-type–bands or lenses in migmatite gneissic terrains; C-type–lenses within alpine-type metamorphic rocks, always coexists with blue schists; Alm–almandine, Grs–grossular, Sps–spessartine, Prp–pyrope. (**e**) X_Mg_ (Mg/(Mg + Fe^2+^)) vs. Si in formula of amphibole. The arrow means the trend of amphibole evolution from inclusion to matrix. See Table S2 for the data employed.

The primary mineral assemblage of eclogite facies is Grt + Omp + Ph + Rt + Qz. The garnet grains are red, subhedral to euhedral, with size ranging from 50 to 4000 μm, and are commonly fractured and filled with retrograde-stage chlorite and epidote (Fig. 2a–d,g). The garnet grains from sample YA-7-18-40 exhibit relatively weak prograde zoning (Alm = 39.8–45.2 mol.%, Sps = 0.7–1.4 mol.%, Prp = 24.2–33.2 mol.%, Grs = 20.6–26.4 mol.%. Table S2; Figs 2c, 3a, S3a), while those from sample YA-7-18-47 show a relatively well-developed prograde-growth core-rim texture (Figs 2d, 3b, S1b,c) with higher pyrope and grossular, lower almandine and spessartine in the rim than in the core (Alm = 47.9–67.0 mol.%, Sps = 0.4–3.4 mol.%, Prp = 6.7–27.6 mol.%, Grs = 14.1–24.2 mol.%. Table S2; Figs 3b, S3b,c). Nevertheless, the core-rim boundary in the garnet grains is obscure or chemically transitional (Fig. 3b). Small (<250 μm) and anhedral inclusions of Ep + Amp + Qz + Rt + Ph commonly exist in garnet core, whereas the garnet rim is relatively free of inclusions (Fig. 2c). The garnet grains are plotted within the C-type eclogite field of ref.^18^, similar to the western CQMB eclogite^4,9,10^ (Fig. 3d).

The omphacite grains are green, subhedral with size ranging from 20 to 200 μm, and inclusion-poor. Jadeite (Jd) contents of these omphacite grains vary from ~39 to ~51 mol.% (Table S2; Fig. 3c). Fractures in the omphacite grains are filled with amphibole and albite, marking breakdown of omphacite during the retrograde metamorphism stage (Fig. 2f).

Based on textural relationships, the amphiboles in the Baqing eclogite may be divided into two groups. One group is the amphibole grains included in garnet grains, and the other group is the replaced amphibole grains that were reaction product of garnet or omphacite in the matrix (Fig. 2a–d,f). According to the amphibole formula of A_0–1_B_2_C_5_T_8_O_22_(OH)_2_^19^, both groups of amphibole grains are Ca or Na–Ca amphibole but not Na amphibole (Table S2). The amphibole grains included in garnet are anhedral and small (<200 μm), while the replaced amphibole grains, mainly barroisite (Brs), are subhedral to anhedral with 10 to 1000 μm in diameter (Fig. 2). The amphibole grains included in garnet exhibit relatively lower SiO_2_ (42–49 wt.%), higher total FeO (11–19 wt.%), and (Na + K)_A_ (molar ratio of [Na + K] in site A, 0.426–0.728), than those of replaced amphibole grains in the matrix (SiO_2_, 47–55 wt.%; total FeO, 8–13 wt.%; (Na + K)_A_, 0.138–0.548) (Table S2; Fig. 3e). However, compositions of both amphibole types overlap each other and are mainly barroisite (Table S2; Fig. 2).

Narrow phengite laths occur in the matrix between garnet, omphacite or amphibole grains, and as inclusions in garnet grains and exhibit Si values of 3.30–3.42 p.f.u. (Table S2; Fig. 2b,c). In general, they appear fresh with no strong deformation. The inclusion phengite grains show lower Si values than the non-inclusion phengite grains (Table S2). Rutile grains occur as inclusion within garnet as well as matrix mineral. They are distinguishable: the former contains more total FeO (~1 wt.%) than the latter (Table S2; Fig. 2a–e). The rutile is partially replaced by ilmenite or titanite (Fig. 2e). Epidote also occurs as inclusion as well as matrix mineral in the Baqing eclogite sample YA-7-18-40 (Fig. 2a,c), while there is less epidote in the eclogite sample YA-7-18-47 (Fig. 2b,d). The epidote compositions are basically coherent with pistacite contents (Fe^3+^/(Al + Fe^3+^)) ranging from 13 to 18 mol.% (Table S2). Similar to amphibole, the inclusion epidote grains are anhedral and smaller (ca. 20 μm) than the matrix epidote (Fig. 2a,c). Only albite rather than plagioclase occurs as the reaction product of omphacite. It disperses in the fractures between different omphacite grains (Fig. 2f). Quartz exists as inclusion in garnet or as matrix around garnet (Fig. 2a–c).

### Major and trace elements

The eight Baqing eclogite samples analyzed have moderate loss on ignition (LOI) values varying from 1.29 to 2.87 wt.% (Table S3). Hereafter we focus on the immobile elements and ratios of specific element pairs, because only these immobile elements could maintain the protolith characteristics under the extreme metamorphic condition^20^. These samples have mafic SiO_2_ (45.73–53.75 wt.%) and Al_2_O_3_ (13.04–14.68 wt.%) contents. In contrast, both CaO (5.61–11.95 wt.%) and MgO contents (4.15–9.29 wt.%) are variable. Furthermore, these samples exhibit high total FeO (11.62–16.94 wt.%) and Na_2_O (2.01–4.00 wt.%), moderate TiO_2_ (1.07–2.41 wt.%), and low K_2_O (0.06–1.04 wt.%) contents.

**Figure 4:**
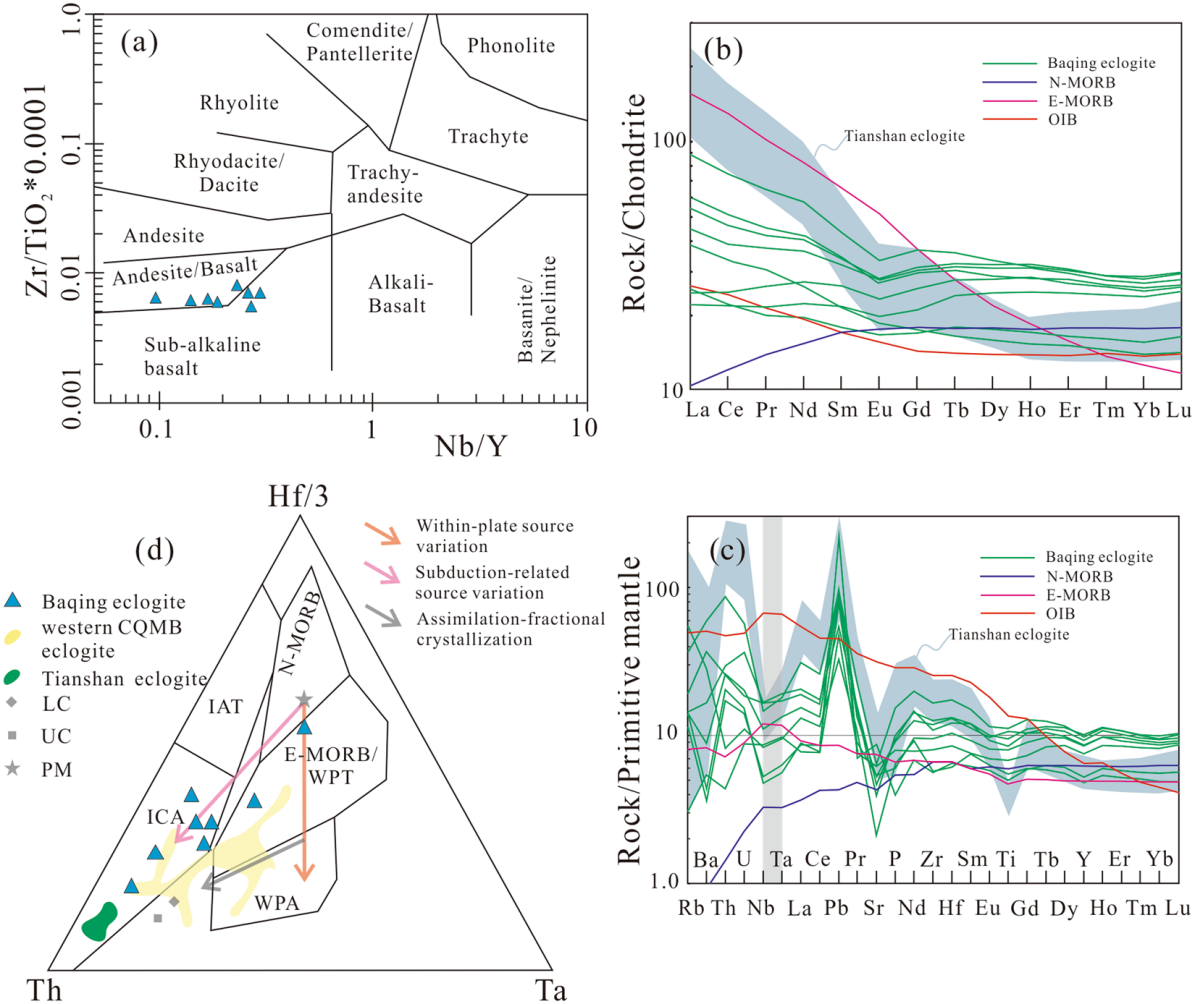
(**a**) Zr/(TiO_2_*0.0001) vs. Nb/Y diagram^60^. The Baqing eclogite samples plotted between the fields of andesite/basalt and sub-alkaline basalt areas. (**b**,**c**) Chondrite-normalized rare-earth element distribution patterns, and Primitive mantle-normalized trace element spider diagrams (The primitive mantle, chondrite, N-MORB, E-MORB and OIB values are from ref.^61^. The Tianshan eclogite data are from ref.^35^). Baqing eclogite shows similar geochemical characteristics to Tianshan eclogite with continental arc origin. They both exhibit LREEs enrichment, flat HREEs pattern, depletion of Nb, Ta, Sr, Ti and enrichment of Pb. (**d**) Hf/3–Th–Ta diagram^62^. The Baqing eclogite samples mainly plotted in the volcanic arc basalt field, along a trend of subduction-related source variation. The western CQMB eclogite data are from ref.^14^. The crustal data are from ref.^63^. The source variation trends are from ref.^36^. The Tianshan eclogite data are from ref.^35^. Abbreviations: WPT–within plate tholeiites; WPA–within plate alkaline; ICA–island arc calc–alkaline basalts; IAT–island arc tholeiites; LC–lower continental crust; UC–upper continental crust; PM–primordial mantle. See Table S3 for the data employed. Figures a–d were drafted by Xin Jin and Yu-Xiu Zhang using the software of CorelDRAW X6.

The Baqing eclogite samples are plotted between the fields of andesite/basalt and sub-alkaline basalt based on immobile elemental ratios (Fig. 4a). These samples have flat ([La/Yb]_N_ = 0.88, 0.93) to enriched ([La/Yb]_N_ = 1.47–3.01) chondrite-normalized rare earth elements (REEs) patterns (Fig. 4b), indicative of slight to moderate enrichments of light REEs (LREEs). These eclogite samples generally exhibit negative Nb and Ta anomalies as well as weakly negative Ti and Y anomalies (Fig. 4c). Additionally, strongly positive Pb and negative Sr anomalies are observed in almost all the samples (Fig. 4c).

### Zircon U–Pb ages and mineral inclusions

**Figure 5:**
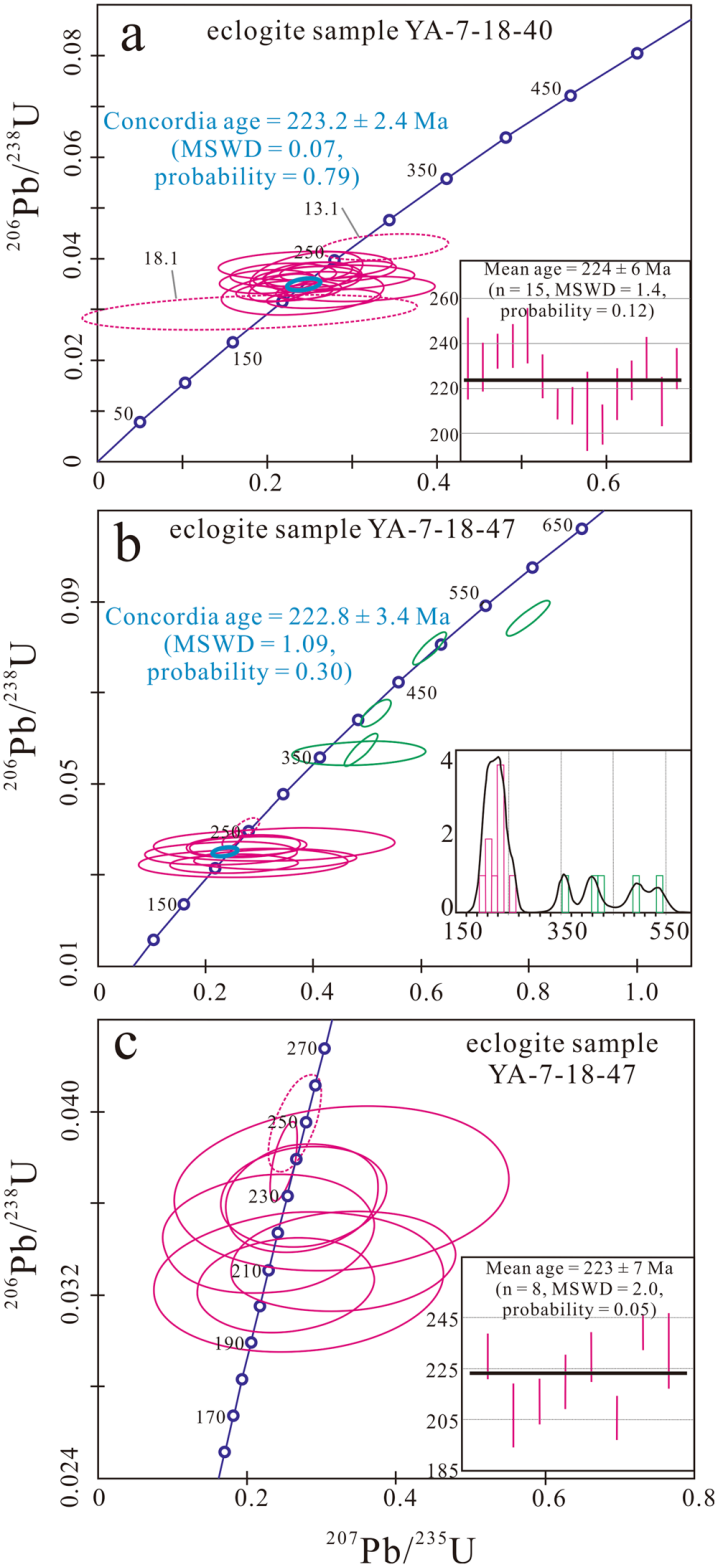
Zircon SHRIMP dating diagrams for the Baqing eclogite. (**a**) Sample YA-7-18-40, (**b**) Sample YA-7-18-47, and (**c**) The detailed metamorphic age of sample YA-7-18-47. Both concordia and weighted mean ages are given. See Table S4 for the data employed.

Zircons collected from two Baqing eclogite samples (YA-7-18-40 and YA-7-18-47) were analyzed using sensitive high-resolution ion microprobe (SHRIMP) II technique (Table S4; Figs 5, S4). The zircons from sample YA-7-18-40 are mainly rounded–ovoid, grey in CL images, and of patch-like structure (Fig. S4a) and are characterized by low Th and U contents (0.02–0.4 ppm and 1.89–42.3 ppm, respectively) and Th/U ratios of 0.002–0.015. Their Th/U ratios are far less than 0.1, typical of a metamorphic origin^21–23^. Among 22 analyzed spots, seven spots were not taken into account for the concordia and weighted mean age calculations based on unreasonable, negative radiogenic ^207^Pb/^206^Pb and ^207^Pb/^235^U ratios, large age error, or older and discrete age (Table S4; Fig. 5a). Fifteen analyses of the metamorphic zircons yielded a concordia age of 223.2 ± 2.4 Ma (mean square of weighted deviation (MSWD) = 0.07) and a weighted mean ^206^Pb/^238^U age of 224 ± 6 Ma (MSWD = 1.5), but the former has much higher probability (0.79) than the latter (0.12) (Fig. 5a).

The zircons from sample YA-7-18-47 can be divided into two groups in terms of their morphologies, internal structures under cathodoluminescence (CL) and Th contents as well as Th/U ratios (Table S4; Fig. S4b). Group 1 zircons are characterized by rounded–ovoid, grey, patch-like structure under CL images, and have dominantly low Th contents of ≤1 ppm and low Th/U ratios of <0.05 (Table S4), indicative of a metamorphic origin^21,22^. Group 2 zircons are characterized by dark inherited core surrounded by narrow grey, metamorphic growth rim (Fig. S4b). Most of these inherited cores display oscillatory zoning and have high Th contents of >200 ppm and high Th/U ratios of >0.4, typical of an igneous origin^21,22^; in contrast, the growth rims are similar to Group 1 zircons in CL image, Th content and Th/U ratios (Fig. S4b). Metamorphic ages were determined based on data obtained from the strictly metamorphic Group 1 zircons, as well as from the metamorphic growth rims of Group 2 zircons. Great care was taken to discriminate inherited zircon cores from metamorphic growth rims, so that the analytical spots could be placed well away from the boundaries between the two zones (Fig. S4b). The inherited zircon cores were easily distinguished from their growth rims by distinctly irregular boundaries, possibly generated by corrosion during metamorphic reworking^21^, which truncate internal zoning and separate the subrounded to irregular cores from the growth rims (Fig. S4b). A total of fourteen zircon spots were analyzed, including seven Group 1 metamorphic zircons, and two narrow growth rims and five inherited cores of Group 2 zircons (Fig. S4b). Five inherited cores yielded variable ages, ranging from the Cambrian to Carboniferous (535 to 355 Ma Fig. 5b). Analyzed spot 4.1 from the growth rim of a Group 2 zircon has obviously mixed with the inherited older core, considering its much higher Th content (6 ppm) than other metamorphic zircons or metamorphic growth rim (≤1 ppm) as well as the narrow width of the rim (Fig. S4b). It yielded a much high ^206^Pb/^238^U age of 256 Ma, and thus was ruled out from the age calculation. Seven Group 1 metamorphic zircons and one metamorphic growth rim of Group 2 zircon yielded a concordia age 222.8 ± 3.4 Ma (MSWD = 1.09), and a weighted mean age of 223 ± 11 Ma (MSWD = 2.0) (Fig. 5b,c),. but the former has much higher probability (0.3) than the latter (0.05) (Fig. 5a).

**Figure 6:**
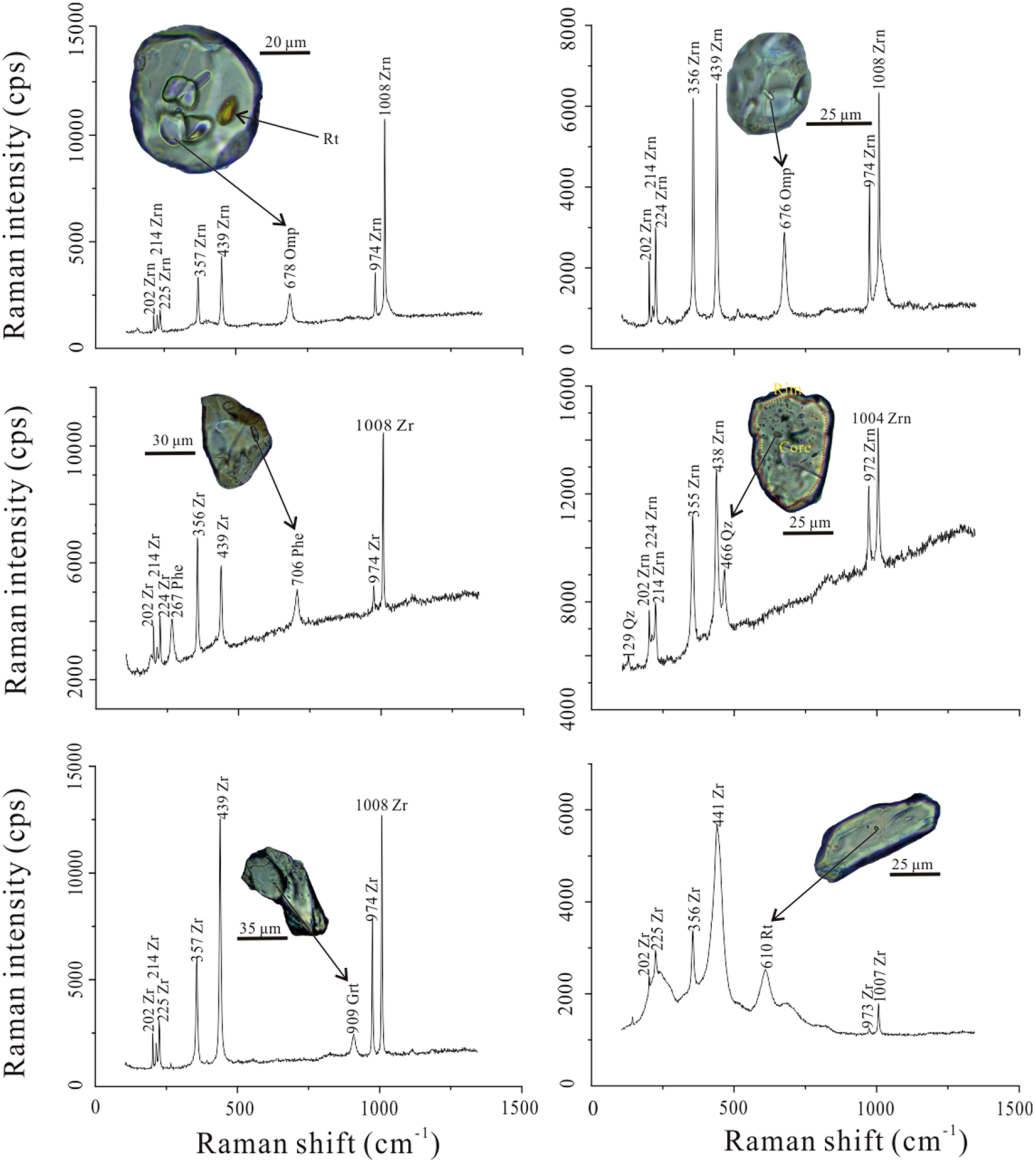
Representative photographs and Raman spectra of zircon inclusions. Omphacite, rutile, garnet and phengite are found in metamorphic zircons in eclogite samples YA-7-18-40 and YA-7-18-47; quartz is found in the cores of inherited zircon. Phengite displays narrow laths in zircon, while other minerals show anhedral. Rutile displays diagnostic yellow.

The inclusions of garnet, rutile, phengite and omphacite were identified in most zircons from sample YA-7-18-40 and Group 1 metamorphic zircon rims of sample YA-7-18-47, quartz inclusions were identified in one inherited Group 2 zircon core (spot 9.1, 535 Ma Fig. 6). The zircon inclusion assemblages (garnet, rutile, phengite and omphacite) are similar with eclogite matrix mineral assemblages, implying that these zircons grew during or shortly after eclogitic facies metamorphism.

Considering the probabilities (0.05 and 0.12) of the two weighted mean ages of the eclogite are too low to be reliable while the probabilities of the two concordia ages (≥0.30; Fig. 5) are quite acceptable^24^, we use the concordia ages of the two eclogites to define the metamorphic age of the Baqing eclogite. The highly concordant relationships between these two age data subsets (223.2 ± 2.4 Ma and 222.8 ± 3.4 Ma) indicate the ages of 223 ± 3 Ma reliably represent the eclogite-phase metamorphic age of the Baqing eclogite.

## Thermobarometry

Combining the garnet zoning, mineral reaction texture and compositional differences, two stages of metamorphic evolution are distinguished: (1) peak metamorphism: Grt (rim) + Omp (matrix) + Ph (matrix) + Rt (matrix) + Qz; and (2) retrograde metamorphism: Amp (matrix) + Ab (matrix) ± Ep (matrix) ± Chl + Ttn (matrix). Considering the overlapping compositions between inclusions in garnet and matrix minerals (Table S2), the minerals in garnet cracks (Fig. 2c,d), and the fluctuant garnet zoning (Fig. 3b), the inclusions in garnet may represent the prograde metamorphism assemblage, subsequently influenced by retrograde metamorphism.

**Figure 7:**
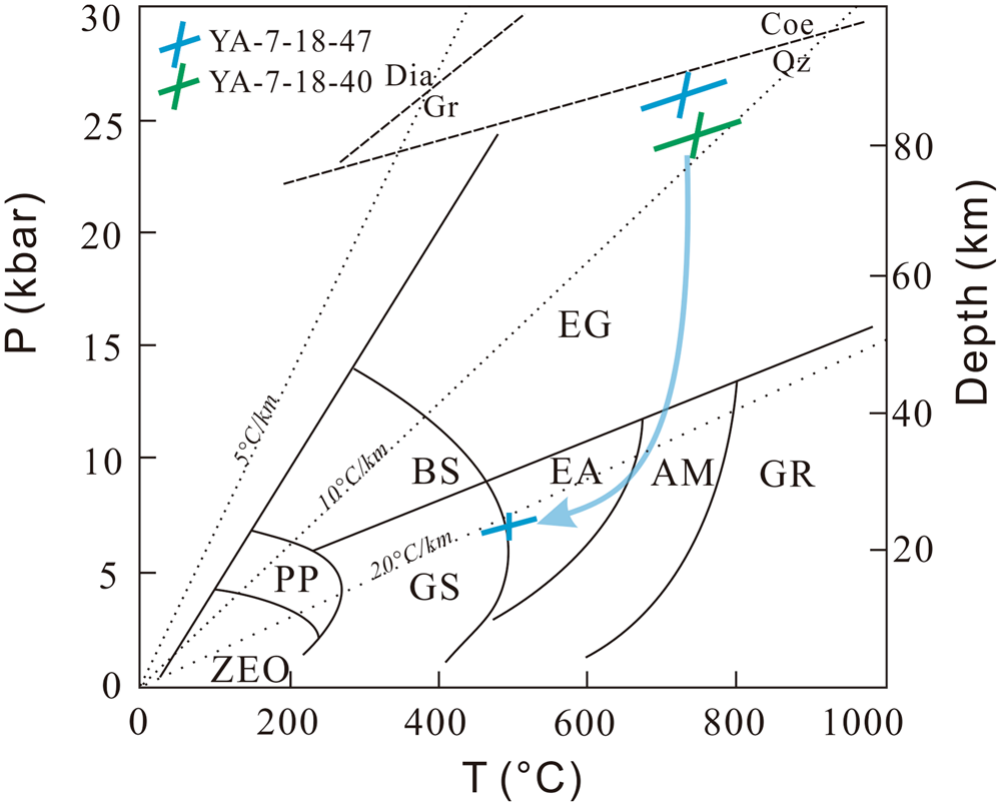
Pressure–temperature diagram showing the peak metamorphic conditions for samples YA-7-18-40 and YA-7-18-47, and retrograde P–T conditions for sample YA-7-18-47. The metamorphic P–T conditions are determined by thermobarometers of Grt–Omp–Ph^26^, Amp–Pl^30^ and Al-in-Amp^31^. The quartz–coesite and diamond–graphite reaction lines are based on refs^64,65^. The grid of metamorphic facies is based on ref.^66^. Abbreviations: EG–eclogite facies; BS–blueschist facies; PP–prehnite–pumpellyite facies; ZEO–zeolite facies; GR–granulite facies; AM–amphibolite facies; EA–epidote amphibolite facies; GS–greenschist facies. Figure was drafted by Xin Jin using the software of CorelDRAW X6.

The omphacite close to the corresponding garnet and of high Jadeite contents, the garnet rim of high pyrope contents (not the outmost rim), and the phengite of high Si contents in the matrix are chosen to mark the peak metamorphic condition. The Grt–Cpx geothermometer^25^ and Grt–Cpx–Ph geobarometer^26^ are appropriate for estimates of the peak metamorphic P–T conditions. These two thermobarometers are based on Grt–Cpx Fe^2+^–Mg cation exchange, and thus exact calibration of Fe^2+^ content is important. We take total Fe in garnet as Fe^2+^ in the calibration since the temperatures estimated by total Fe are indistinguishable to those by Fe^2+^ obtained by Mossbauer spectrum^27^. Fe^3+^ in omphacite is estimated using the charge balance method of ref.^28^. The peak metamorphism is estimated at conditions of 25 ± 1 kbar/730 ± 60 °C (Fig. 7).

The retrograde metamorphism is marked by titanite as corona around rutile, chlorite and epidote that replaced garnet (Fig. 2d,g), and symplectite of amphibole and albite replacing omphacite at the rim (Fig. 2c). The garnet outmost rim shows a little composition variation (Figs 3b, S3b), indicative of a retrograde metamorphic progress. These minerals may originate from the reaction of Omp + Grt + Rt + H_2_O = Amp + Ab + Ep + Ttn, an important boundary reaction between epidote amphibolite and eclogite facies^29^. Based on the retrograde mineral assemblages, Amp + Pl geothermometer^30^ and Al-in-Amp barometer^31^ were used. The retrograde metamorphic pressure and temperature are estimated to be 7 ± 0.6 kbar at 480 ± 35 °C (Fig. 7).

## Discussion

### Continental arc-related protolith of the Baqing eclogite

The relatively moderate Mg contents of garnet (X_Mg_ = Mg/(Mg + Fe^2+^)) are from 0.09 to 0.43, on average, 0.34 (Table S2), compared to mantle eclogite (X_Mg_ = 0.78–0.93)^4,32^, imply that the Baqing eclogite is unlikely of mantle origin, consist with the orogenic origin of the C-type eclogite field^18^ (Fig. 3c). Moreover, the mantle eclogite surrounded by ultramafic rock always displays high metamorphic temperature and Mg contents^32^. Compared to mantle eclogite^32^, Baqing eclogite is unlikely of mantle origin. Their moderate TiO_2_ contents (Table S3) are distinct from the high TiO_2_ contents of the western CQMB eclogite with protolith of continental flood basalt or OIB^4,10^. The low TiO_2_ contents of the Baqing eclogite are similar to those of normal mid-ocean ridge basalt (N-MORB) and arc-related basalt^33^. Their LREE enrichments (Fig. 4b) indicate that their protoliths should not be depleted or N-MORB, and the negative Nb–Ta anomalies (Fig. 4c) indicate that their protoliths are contaminated by crustal material and thus formed most likely in a continental arc-related environment^33^. It is also supported by the strongly positive Pb and negative Sr anomalies as depleted Sr and enriched Pb is widespread in upper continental crust^34^. These characteristics are similar to the Tianshan eclogite with continental arc origin (Fig. 4b,c). In Hf/3–Th–Ta triangular discrimination diagram, the Baqing eclogite samples were mainly plotted in volcanic arc basalt field, along a trend of subduction-related source variation, which is different from the trend of western CQMB eclogite^34^ (Fig. 4d). Importantly, the presence of Paleozoic zircon cores with high Th/U ratios (>0.1. Table S4) indicates they were inherited zircons with a magmatic origin^21,22^. The disperse ages of zircon cores indicate that they were captured in the process of upper-continental crust contamination. The protoliths of the Baqing eclogite most likely represent continental arc-related basites erupted in active continental margin, similar to the Tianshan eclogite with continental arc affinities^35^, although the formation age of the protoliths is obscure due to limited SHRIMP zircon dating and the lack of zircon in the basaltic protolith.

When the juvenile and hot arc subducted into mantle, the arc-related basites will be metamorphosed into eclogites with high geothermal gradient (hot eclogite)^36^. This may explain the high geothermal gradient (~9 °C/km) of the Baqing eclogite. Furthermore, amphibole with different chemical compositions coexisting with eclogitic minerals represents different associated geothermal gradients^37,38^, with Na amphibole (e.g. glaucophane) corresponding to cold eclogites, Ca amphibole (e.g. hornblende) to hot eclogites, and Na–Ca amphibole (e.g. barroisite) to Ep-amphibolites. The Ca amphibole of the Baqing eclogite is consistent with high geothermal gradient, distinctly higher than that of the western CQMB eclogite (~6 °C/km)^4,9^.

The Triassic continental arc is located in the southern margin of the East Qiangtang subterrane north of the Baqing eclogite-bearing metamorphic belt (Fig. 1b). It has been found that the crustal materials with the overriding East Qiangtang affinity were involved into the Shuanghu Paleo-Tethyan subduction zone and were then exhumed in the western Qiangtang region^39^. Moreover, studies on the Tianshan eclogite indicate that it was product of subduction of matters eroded from overriding continental arc^35^. Likewise, we suppose the Baqing eclogite originated from subduction of the materials eroded from the East Qiangtang continental arc during the Late Triassic.

### Correlation of the Baqing Eclogite to the Shuanghu Paleo-Tethyan suture

The Bangong suture zone is characterized by Jurassic high-pressure metamorphism, including, from west to east, Gaize eclogite or amphibolite (194–170 Ma)^40^, Anduo granulite-facies metamorphism (190–178 Ma)^41–43^, Basu eclogite or orthogneiss (173 Ma)^44,45^ (Figs 1, S5). Close to north of the Bangong Meso-Tethyan suture zone and along southern margin of West Qiangtang subterrane, exists Jurassic (–Lower Cretaceous) arc magmatism^13,46^ (Fig. 1b). This Bangong Meso-Tethyan branch did not close until the Mid-Cretaceous based on radiolarian-bearing ophiolitic fragments and arc-related magmatic records^13,47^. By contrast, however, the eastern CQMB contains Triassic flysch, ophiolite fragments^1,47^ and Triassic tectonic schist^16^, and a Permian–Triassic magmatic arc is juxtaposed close to its north in the East Qiangtang subterrane^48^ (Fig. 1b). The Baqing eclogite is within the eastern CQMB and can be tectonically and temporally correlated with the western CQMB eclogite, despite of the different protoliths^4,9,14^ (Figs 1b, 4d). Furthermore, the high-pressure metamorphic ages (Late Triassic: 223 Ma) of the Baqing eclogite in the eastern CQMB are much older than those (Early–Middle Jurassic: 194–170 Ma) of the Bangong Meso-Tethyan suture zone (Fig. 1b). Apparently, the eclogite-bearing eastern CQMB represents the relict of a Paleo-Tethyan branch that closed during the Late Triassic^10,47^ and is most likely correlated with the Shuanghu suture^10^. Therefore, we believe that the Baqing eclogite is not correlated with the Bangong Meso-Tethyan suture but the Shuanghu suture.

### Anticlockwise collision between the West and East Qiangtang subterranes along the Shuanghu Paleo-Tethyan suture

**Figure 8:**
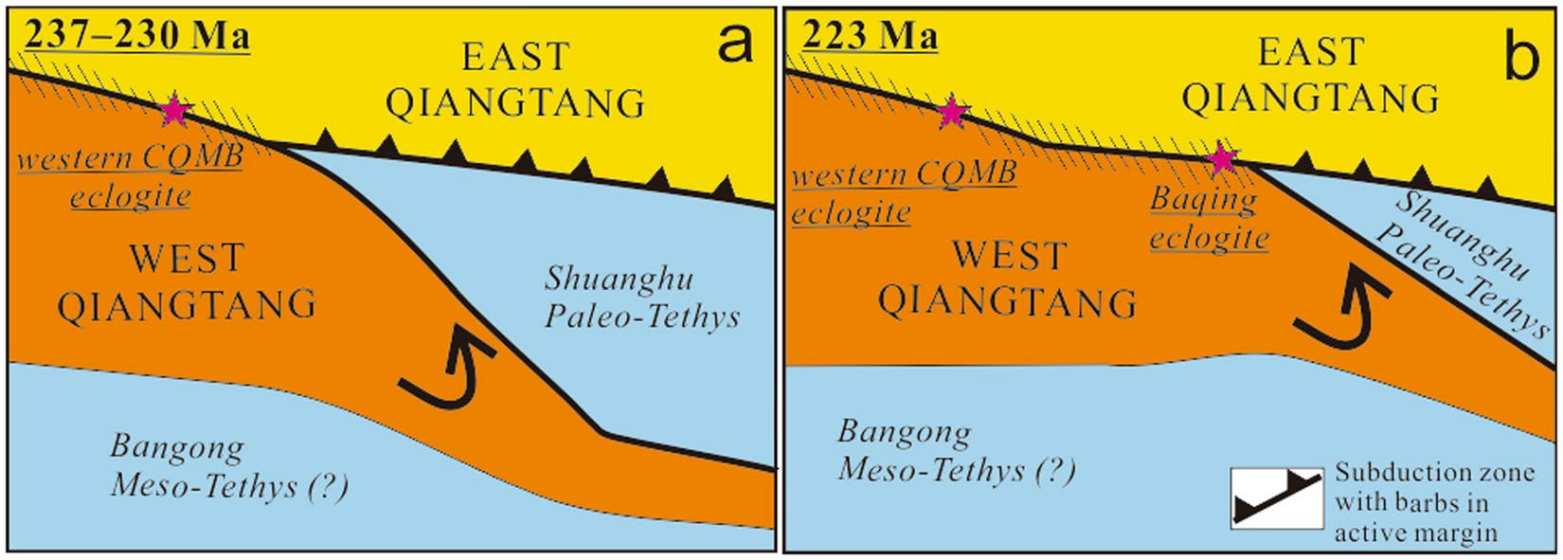
Schematic overview of the eastward-younging anticlockwise collision between the West and East Qiangtang subterranes during the Triassic, as indicated by the western CQMB and Baqing ecloigtes.

Identification of the Late Triassic Baqing eclogite in this study confirms both the eastern extension of the Late Triassic CQMB and thus the existence of the Shuanghu Paleo-Tethyan suture that separates the West and East Qiangtang subterranes (Fig. 8). To the east of the Baqing eclogite along the eastern CQMB are exposed the Dingqing Triassic ophiolite fragments^49^, bedded Triassic radiolaria-bearing cherts, trench Triassic turbidites^1,47^, and arc-related magmatic rocks^48^, as well as Late Triassic deformation zone^16^, together supporting the existence of the Triassic Shuanghu suture zone (Fig. 1b). The Baqing eclogite thus provides vital constraints on the tectonothermal evolution of the Paleo-Tethys.

Across the CQMB, the collision between the West and East Qiangtang terranes occurred during the Middle Triassic, as evidenced by dating (237–230 Ma) of the western CQMB continental eclogite^4,9,10^ (Fig. 8a). The West and East Qiangtang continental collision in eastern Qiangtang, marked by dating of the Baqing eclogite of this study, is ~10 Ma younger than those in western Qiangtang, showing a scissor-like, eastward-younging, anticlockwise collision between these two continental subterranes (Fig. 8). This model is also supported by the 217-Ma Dingqing ophiolites, east of the Baqing area^48^ (Figs 1b, 8).

## Conclusions

(1) The newly discovered Baqing eclogite has eclogite-facies peak metamorphic mineral assemblage of Grt + Omp + Rt + Ph + Qz, and formed at metamorphic conditions of 25 ± 1 kbar/730 ± 60 °C. The Ep-amphibolite facies retrograde metamorphism formed at condition of 7 ± 0.6 kbar/480 ± 35 °C. (2) The Baqing eclogite exhibits LREE enrichments, negative Nb–Ta anomalies, weak negative Ti anomalies, and Paleozoic inherited zircon cores, marking a continental magmatic arc origin. (3) The inclusions of Grt + Omp + Rt + Ph in zircon grains imply that they experienced eclogitic phase metamorphism. The zircon SHRIMP U–Pb dating indicates the peak eclogite metamorphism occurred at 223 Ma. (4) The Baqing eclogite was formed by the scissor-like, eastward-younging anticlockwise collision between the West and East Qiangtang subterranes along the Shuanghu Paleo-Tethyan suture during the Late Triassic.

## Methods

### Mineral chemistry analyses

Mineral compositions and X-ray compositional mapping of garnets were determined using a JOEL JXA 8100 electron microprobe equipped at the Institute of Geology and Geophysics, Chinese Academy of Sciences (IGGCAS), Beijing, China. Mineral compositions are operated at 15-kV accelerating voltage, 20-nA beam current, 10-second counting time, and 5-μm electron beam diameter on the minerals. The detection limit is 0.01 wt.% for all the analyzed elements. Synthetic and natural minerals were used as standards (albite (Na), diopside (Si, Ca), periclase (Mg), hematite (Fe), orthoclase (K), rhodonite (Mn), synthetic Cr_2_O_3_ (Cr), synthetic TiO_2_ (Ti), and synthetic Al_2_O_3_ (Al). The program ZAF was used for matrix corrections^50^. In garnet compositional profiles (Fig. 2c,d), inclusions and cracks in garnets were avoided. Results of representative mineral compositions for the Baqing eclogite are shown in Table S2.

### Major and trace element analyses

Whole-rock major and trace element analyses were performed at the Modern Analysis Center, Nanjing University, Nanjing, China, and the IGGCAS, respectively. Major element oxides were analyzed on wavelength-dispersive X-ray fluorescence spectrometry (ARL9800+) using fused glass pellets. Analytical precision determined through replicate analyses is better than 0.5%. Trace elements (including REEs) were analyzed using an Inductively Coupled Plasma–Mass Spectrometry (ICP–MS) (Element, Finnigan MAT) with solution methods. The analytical precision determined through replicate analyses is within 5–10% for all trace elements. Results of major and trace elements are shown in Table S3.

### Zircon SHRIMP U–Pb geochronology analyses

Zircon grains from Baqing eclogite (samples YA-7-18-47 and YA-7-18-40) were separated using standard heavy liquid and magnetic methods. Photomicrographs of zircon grains under transmitted and reflected light, and cathodoluminescence (CL) images under Hitachi S3000N SEM were obtained at the Beijing SHRIMP Center, China, in order to reveal the internal structures of the grains and to select target sites. The U, Th, and Pb contents of zircons were measured using SHRIMP II at the Beijing SHRIMP Center, China, under standard operating conditions (15 kV accelerating voltage and a 20-nA beam current). The U–Th–Pb ratios and the absolute abundances of U and Th were determined relative to the standard zircons TEMROA and SL13^51^. Measured compositions were corrected for common Pb using non-radiogenic ^204^Pb (sample YA-7-18-47) and ^208^Pb (sample YA-7-18-40) based on the different genesis of zircons, and an average crustal composition^52^ appropriate for the age of the mineral was assumed. Errors on individual analysis are based on counting statistics at one standard deviation (1σ) level. The weighed mean ^206^Pb/^238^U age data are quoted at 95% confidence level^53^. Results of zircon U–Pb dating data are shown in Table S4.

### Zircon inclusion analyses

In order to define that the zircons or zircon rims were growing as peak eclogite facies mineral assemblage formed, zircon inclusions were identified by a laser Raman microspectrophotometer (Renishaw, UK: inVia Reflex) at School of Earth and Space Sciences, Peking University, Beijing, China. It is equipped with a 532 nm DPSS Laser. The Raman spectra are from 100 cm^−1^ to 1350 cm^−1^ and the wave-number accuracy is better than 1 cm^−1^. The XY and Z resolutions were about 0.5 μm and 2 μm, respectively, with a 2400 lines/mm grating and confocal mode using a Leica 100 × /0.85 micro-objective. The laser spot power on the surface was set to ~2 mW. The temperature in experimental room was about 22 °C.

## Electronic supplementary material

Supplementary information
