# Supplementary material for: Newly discovered Late Triassic Baqing eclogite in central Tibet indicates an anticlockwise West–East Qiangtang collision
Source: Sci Rep. 2018 Jan 17;8:966. doi: 10.1038/s41598-018-19342-w (PMC5772616; doi:10.1038/s41598-018-19342-w)
Supplement: Supplementary file 1 — Supplementary information [file 41598_2018_19342_MOESM1_ESM.pdf]

Newly discovered Late Triassic Baqing eclogite in central Tibet indicates an anticlockwise West– East Qiangtang collision

Yu-Xiu Zhang [1, 2], Xin Jin [1], Kai-Jun Zhang [1, 2, *], Wei-Dong Sun [3, 4], Jian-Ming Liu [1], Xiao-Yao Zhou [1], Li-Long Yan [1]

[1.] Asian Tectonics Research Group, College of Earth Science, University of Chinese Academy of Sciences, 19A Yuquan Road, Beijing 100049, China;

[2.] Key Laboratory of Computational Geodynamics, Chinese Academy of Sciences, 19A Yuquan Road, Beijing 100049, China;

[3.] Center of Deep Sea Research, Institute of Oceanology, Chinese Academy of Sciences, Qingdao 266071, China;

[4.] CAS Center for Excellence in Tibetan Plateau Earth Sciences, Chinese Academy of Sciences, Guangzhou 510640, China

[*] Correspondence and requests for materials should be addressed to K.J. Zhang (E-mail: kaijun@ucas.ac.cn)

Online supplementary information

Includes the following materials:

1. Supplementary figures.

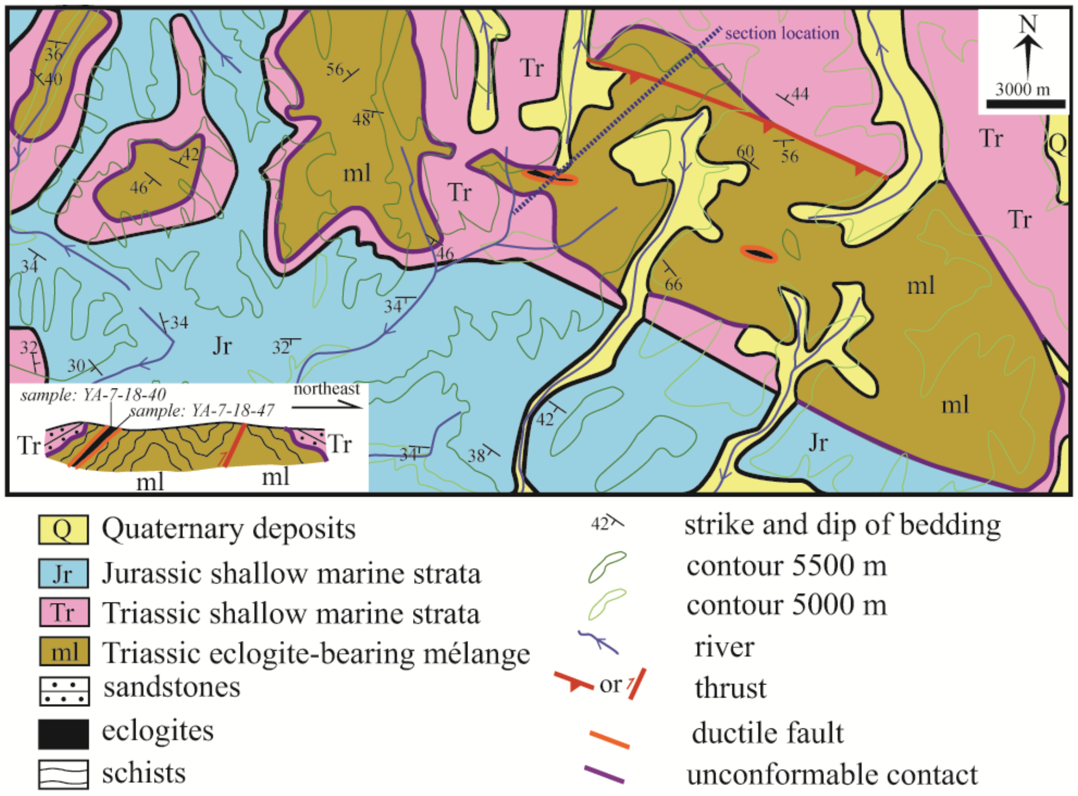

Figure S1. Geologic map and cross section of the study area.

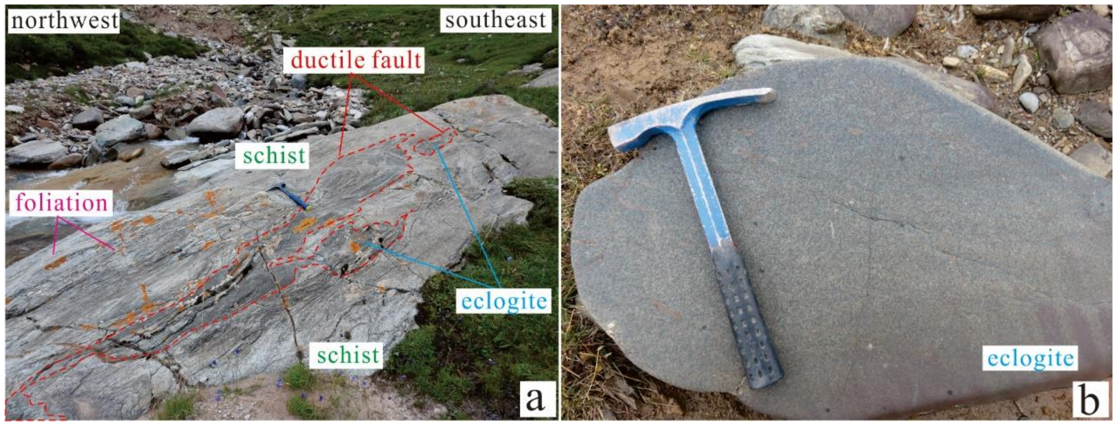

Figure S2. (a, b) Field pictures of eclogite, foliated garnet-mica-quartz schist and ductile fault. Eclogite ocuurs as lenses/boudins in the host garnet-mica-quartz schists

with size ranging from 0.5 to 3 m. They are ductile fault contact. Eclogite boudins are parallel to dominant foliation in the garnet-mica-quartz schists. The dominant foliation strike in the garnet-mica-quartz schists is in northwest–southeast direction. Hammer for scale.

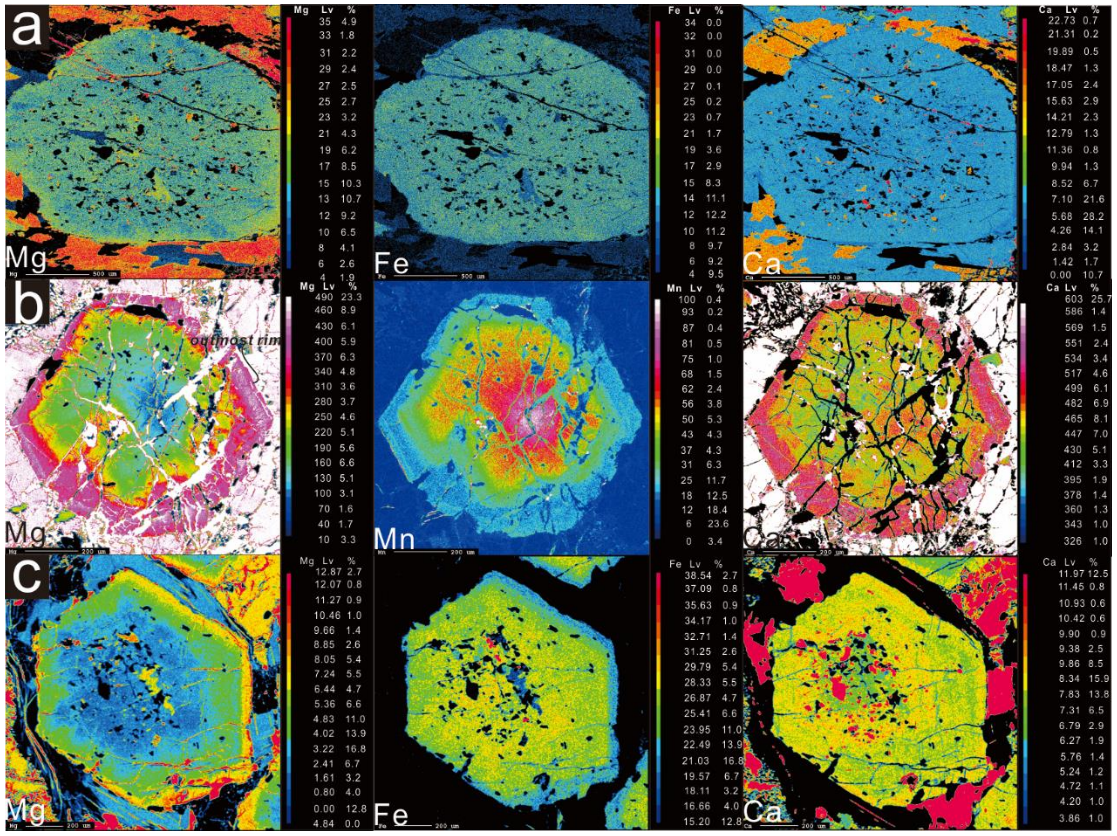

Figure S3. Garnet compositional element maps for eclogite samples YA-7 -18-40 and YA-7-18-47. (a) Mg, Fe, Ca maps for garnet from sample YA-7-18-40, showing weak zoning, Mg and Ca contents increasing and Fe contents decreasing from core to rim. It is consistent with the result of the garnet compositional profiles (Fig. 3a). (b) Mg, Mn, Ca maps for garnet from sample YA-7-18-40, showing obvious zoning, Mg and Ca contents increasing and Fe contents decreasing from core to mantle, then to rim. The outmost rim is opposite to this trend. It is consistent with the result of garnet compositional profiles (Fig. 3b). (c) Mg, Fe, Ca maps for garnet from sample YA-7-18-47, showing obvious zoning, Mg and Ca contents increasing and Fe contents decreasing from core to rim.

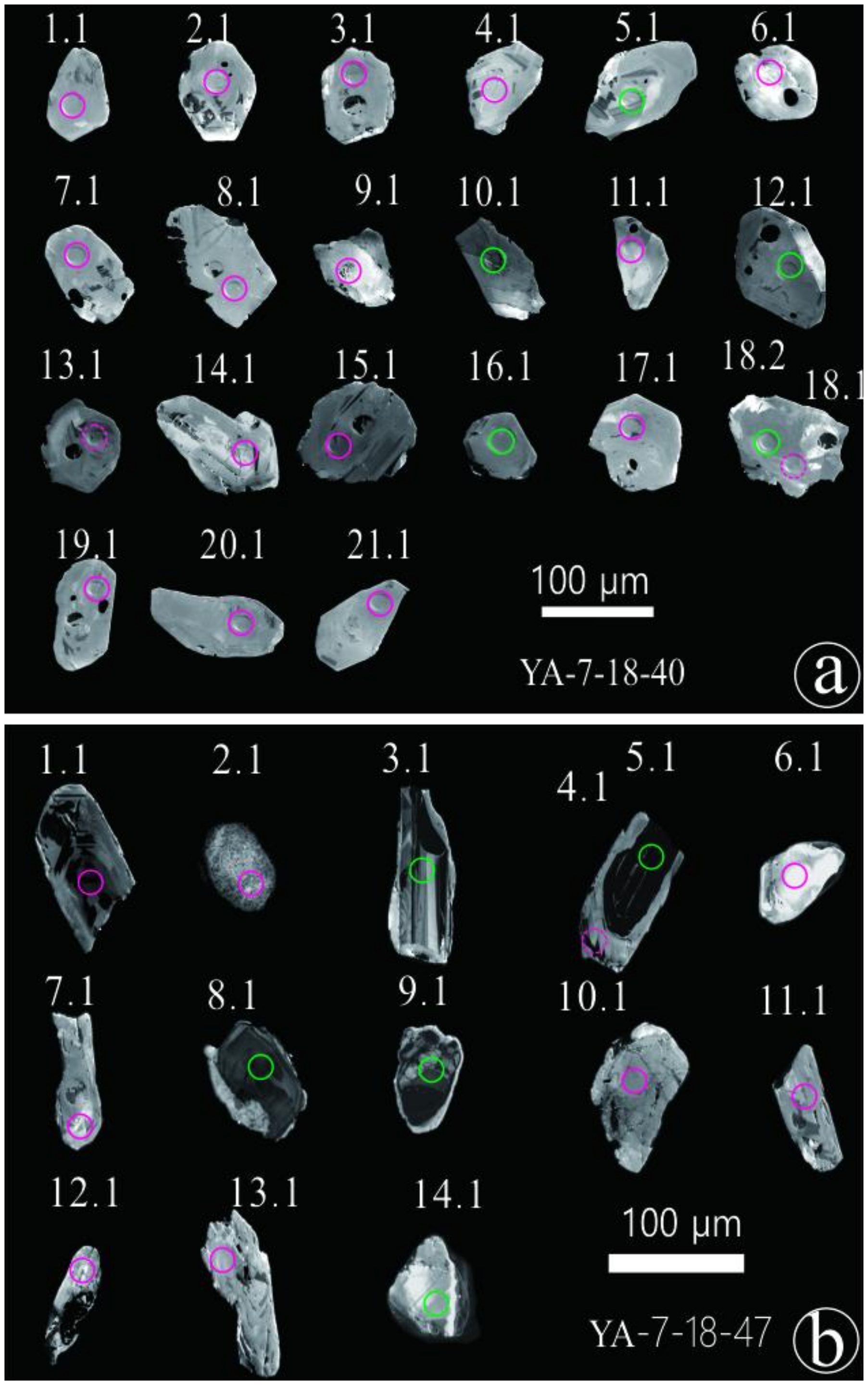

Figure S4. Zircon cathodoluminescence (CL) images. (a) The fifteen red solid spots show the metamorphic zircons employed in the age calculation of YA-7-18-40 (Fig. 5a). These five green spots with wrong radiogenic $^{207}Pb/^{206}Pb$ and $^{207}Pb/^{235}U$ ratios and the dotted red spots with high age error, are not involved in the concordia and weighted mean $^{206}Pb/^{238}U$ age calculation. (b) In sample YA-7-18-47, the five green circles show the zircon dark older cores, which, along with the spot 4.1 (rim), are not involved in the concordia and weighted mean $^{206}Pb/^{238}U$ age calculation (Fig. 5b). The other 8 red spots of metamorphic zircons or metamorphic growth rim are used in age calculation of Fig. 5b.

2. Supplementary tables of mineral geochemical, geochemical, and geochronological data.

Table S1. Metamorphic mineral assemblages for Baqing eclogite.

Eclogite sample	YA-7-18-40	YA-7-18-47
Inclusions in garnet	Amp + Ep + Ph + Qz + Rt	Qz + Rt + Ep
Matrix minerals	Grt + Omp + Ph + Rt + Qz + Amp ± Ab + Ep	Grt + Omp + Ph + Rt + Qz +Amp +Ep + Ab
Secondary assemblages (Possible)	Chl + Ep + Amp ± Ab + Ttn/Ilm	Chl +Amp + Ep + Ab + Ttn/Ilm

Table S2. Electron microprobe analyses (wt.%) and positive ion calculation.

Eclogite sample												
YA-7-18-47						YA-7-18-40						
No.	Line 1	Line 13	Line 21	Line 34	Line 35	Line 1	Line 2	Line 13	Line 14	Line 21	Line 34	Line 35
Mineral	Grt (outmost)	Grt (mantle)	Grt (core)	Grt* (rim)	Grt (outmost)	Grt (outmost)	Grt (rim)	Grt (core)	Grt (core)	Grt (mantle)	Grt* (rim)	Grt (outmost)
SiO_2	38.84	38.65	37.23	39.19	39.78	40.12	40.13	39.29	39.59	40.11	39.63	39.03
TiO_2	0.02	0.13	0.13	0.00	0.01	0.00	0.04	0.15	0.09	0.12	0.04	0.02
Al_2O_3	21.78	21.47	20.71	22.17	21.98	22.62	22.65	21.95	21.54	22.02	22.30	22.74
TFeO	22.52	26.91	29.52	22.37	23.11	21.18	19.47	21.58	21.35	20.87	20.50	19.54
Cr_2O_3	0.00	0.02	0.03	0.02	0.02	0.07	0.07	0.04	0.04	0.07	0.06	0.06
MnO	0.22	0.77	1.48	0.19	0.23	0.37	0.34	0.59	0.61	0.54	0.40	0.33
MgO	6.61	4.63	1.69	7.23	6.79	8.88	8.33	7.50	7.20	8.28	7.92	8.39
CaO	8.60	7.27	7.79	8.55	8.33	7.76	9.93	9.26	9.84	8.60	9.88	9.72
Na_2O	0.08	0.03	0.18	0.01	0.08	0.03	0.02	0.04	0.03	0.15	0.00	0.04
K_2O	0.00	0	0.02	0.00	0.00	0.00	0.01	0.00	0.00	0.00	0.02	0.06
Total	98.67	99.86	98.78	99.72	100.34	101.02	100.99	100.42	100.29	100.77	100.74	99.88
O	24.00	24.00	24.00	24.00	24.00	24.00	24.00	24.00	24.00	24.00	24.00	24.00

Si	6.039	6.0493	6.025	6.014	6.077	6.021	6.016	5.992	6.048	6.053	5.993	5.932
Al	3.990	3.961	3.951	4.009	3.959	4.001	4.002	3.946	3.879	3.917	3.975	4.073
Ti	0.002	0.016	0.015	0.000	0.001	0.000	0.005	0.017	0.010	0.013	0.005	0.002
Cr	0.000	0.000	0.004	0.002	0.003	0.008	0.008	0.005	0.005	0.009	0.007	0.007
Fe^{2+}	2.928	3.522	3.995	2.870	2.952	2.659	2.441	2.753	2.727	2.634	2.593	2.484
Fe^{3+}	0.000	0.000	0.000	0.000	0.000	0.000	0.000	0.000	0.000	0.000	0.000	0.000
Mn	0.029	0.101	0.202	0.024	0.030	0.046	0.043	0.076	0.079	0.070	0.051	0.042
Mg	1.533	1.079	0.407	1.654	1.546	1.987	1.682	1.705	1.639	1.863	1.785	1.894
Ca	1.432	1.219	1.351	1.406	1.363	1.248	1.594	1.514	1.610	1.390	1.600	1.583
Na	0.025	0.008	0.058	0.002	0.023	0.008	0.006	0.011	0.009	0.043	0.000	0.010
K	0.000	0.000	0.004	0.000	0.000	0.000	0.002	0.000	0.000	0.000	0.003	0.011
Cations	15.977	15.958	16.014	15.982	15.954	15.978	15.979	16.021	16.005	15.993	16.012	16.037
X_{Py}	0.259	0.182	0.068	0.278	0.262	0.334	0.314	0.282	0.271	0.313	0.296	0.316
X_{Alm}	0.494	0.595	0.671	0.482	0.501	0.448	0.411	0.455	0.450	0.442	0.430	0.414
X_{Grs}	0.242	0.206	0.227	0.236	0.231	0.210	0.268	0.250	0.266	0.233	0.265	0.264
X_{Sps}	0.005	0.017	0.034	0.004	0.005	0.008	0.007	0.013	0.013	0.012	0.008	0.007

Sample	YA-7-18-47													
No.	1	1-O	2	2-E	3	3-O	5	7	8	8.2	10	10.1	11	12
Mineral	Omp	Omp	Omp	Omp	Omp*	Omp	Omp	Omp	Omp	*Omp*	Omp	Omp	Omp	Omp
SiO_2	57.41	56.86	57.77	56.99	57.41	57.19	57.13	57.81	57.30	57.22	57.40	57.17	57.80	57.88
TiO_2	0.08	0.08	0.09	0.05	0.07	0.06	0.06	0.05	0.07	0.06	0.04	0.03	0.04	0.01
Al_2O_3	11.41	11.19	11.63	10.80	11.45	11.84	11.22	11.49	10.55	11.82	9.91	11.44	11.57	10.07
TFeO	4.09	3.95	3.88	4.98	3.89	3.77	3.93	3.92	5.03	3.77	6.36	4.65	3.83	5.95
Cr_2O_3	0.01	0.00	0.00	0.02	0.02	0.02	0.02	0.03	0.00	0.02	0.01	0.00	0.01	0.00
MnO	0.01	0.00	0.03	0.00	0.02	0.00	0.00	0.00	0.01	0.02	0.00	0.06	0.00	0.00
MgO	8.16	8.12	8.08	7.63	7.98	7.82	8.33	8.01	8.26	7.92	7.74	8.58	7.97	8.15

CaO	13.01	12.84	12.82	12.28	12.89	12.44	13.00	12.43	12.85	12.58		12.23	12.73	12.56	13.08
Na_2O	7.23	7.06	7.14	7.60	7.27	7.37	7.23	7.46	7.39	7.54		7.35	7.02	7.43	7.21
K_2O	0.01	0.02	0.00	0.01	0.00	0.00	0.00	0.00	0.01	0.00		0.00	0.06	0.00	0.02
Total	101.41	100.12	101.47	100.40	101.00	100.51	100.94	101.22	101.48	100.95		101.07	101.77	101.23	102.38
O	6.00	6.00	6.00	6.00	6.00	6.00	6.00	6.00	6.00	6.00		6.00	6.00	6.00	6.00
Si	2.007	2.011	2.012	2.020	2.012	2.010	2.006	2.019	2.012	2.005		2.032	1.996	2.018	2.022
Al	0.470	0.467	0.478	0.451	0.473	0.491	0.464	0.473	0.437	0.488		0.414	0.471	0.476	0.415
Ti	0.002	0.002	0.002	0.001	0.002	0.002	0.002	0.001	0.002	0.002		0.001	0.001	0.001	0.000
Cr	0.000	0.000	0.000	0.001	0.001	0.000	0.001	0.001	0.000	0.000		0.000	0.000	0.000	0.000
Fe^{2+}	0.117	0.117	0.113	0.107	0.114	0.111	0.098	0.114	0.057	0.096		0.151	0.123	0.112	0.131
Fe^{3+}	0.003	0.000	0.000	0.040	0.000	0.000	0.017	0.000	0.090	0.014		0.037	0.013	0.000	0.042
Mn	0.000	0.000	0.001	0.000	0.001	0.000	0.000	0.000	0.000	0.000		0.000	0.002	0.000	0.000
Mg	0.425	0.428	0.420	0.403	0.417	0.410	0.436	0.417	0.433	0.414		0.408	0.446	0.414	0.424
Ca	0.487	0.487	0.479	0.466	0.484	0.468	0.489	0.465	0.484	0.472		0.464	0.476	0.470	0.490
Na	0.490	0.484	0.482	0.522	0.494	0.502	0.492	0.505	0.503	0.512		0.505	0.475	0.503	0.489
K	0.000	0.001	0.000	0.000	0.000	0.000	0.000	0.000	0.000	0.000		0.000	0.003	0.000	0.001
Cations	4.001	3.996	3.987	4.014	3.996	3.994	4.006	3.996	4.019	4.005		4.013	4.007	3.995	4.015
X_{Jd}	0.481	0.481	0.497	0.457	0.484	0.505	0.473	0.487	0.442	0.496		0.427	0.495	0.489	0.390

Omp in italic decomposed into Amp + Ab.

Sample	YA-7-18-40				YA-7-18-47							YA-7-18-40			
No.	12	3	6.1	11	6	13	8.3	0	2	8.1	11	1	1-O	2	3	4
Mineral	Omp*	Ph (inclusion)	Ph[*]	Ph	Ph	Ph[*]	Ab[&] (Omp decomposed)	Magnesiokatophorite	Barroisite	Edenite[&] (Omp decomposed)	Barroisite	Barroisite (inclusion)	Barroisite	Barroisite	Barroisite	Barroisite
SiO_2	56.46	46.97	50.70	49.72	50.54	52.21	68.63	47.18	49.35	47.79	52.59	46.17	52.55	50.12	52.43	51.79
TiO_2	0.11	0.34	0.38	0.32	0.33	0.33	0.01	0.21	0.16	0.12	0.09	0.15	0.15	0.23	0.16	0.18
Al_2O_3	8.95	27.34	28.02	27.54	27.35	27.82	19.38	13.77	12.70	12.70	9.71	12.66	7.96	10.19	8.81	9.19
TFeO	4.23	2.01	1.96	1.98	1.53	1.55	0.31	13.22	10.23	11.23	7.63	15.91	9.68	10.89	9.43	9.80
Cr_2O_3	0.08	0.01	0.03	0.01	0.05	0.09	0.00	0.03	0.02	0.01	0.03	0.06	0.03	0.14	0.13	0.03

MnO	0.04	0.01	0.03	0.02	0.00	0.00	0.02	0.06	0.05	0.07	0.01	0.21	0.10	0.10	0.06	0.05
MgO	10.37	3.23	3.16	3.08	3.49	3.62	0.09	11.10	12.78	12.23	14.61	9.75	15.12	13.34	14.57	14.54
CaO	16.49	0.06	0.01	0.04	0.03	0.01	0.87	9.66	8.89	10.48	7.02	9.45	9.32	9.55	9.31	9.49
Na_2O	4.85	0.74	0.74	0.75	0.68	0.69	11.51	3.78	3.69	3.17	4.06	3.34	2.96	3.16	3.15	3.26
K_2O	0.00	9.88	10.15	10.08	9.90	10.01	0.06	0.48	0.50	0.31	0.20	0.37	0.22	0.27	0.25	0.28
Total	101.59	90.59	95.22	93.52	93.91	96.35	100.89	99.49	98.40	98.11	95.97	98.07	98.08	98.04	98.35	98.67
O	6.00	11.00	11.00	11.00	11.00	11.00	8.00	23.00	23.00	23.00	23.00	23.00	23.00	23.00	23.00	23.00
Si	1.988	3.30	3.38	3.37	3.40	3.42	2.983	6.74	6.98	6.85	7.45	6.78	7.41	7.14	7.37	7.28
Al	0.372	2.27	2.20	2.20	2.17	2.15	0.993	2.32	2.12	2.15	1.62	2.19	1.32	1.71	1.46	1.52
Ti	0.003	0.02	0.02	0.02	0.02	0.02	0.000	0.02	0.02	0.01	0.01	0.02	0.02	0.02	0.02	0.02
Cr	0.002	0.00	0.00	0.00	0.00	0.00	0.000	0.00	0.00	0.00	0.00	0.01	0.00	0.02	0.01	0.00
Fe^{2+}	0.125	0.06	0.05	0.06	0.04	0.04	0.011	0.10	1.09	1.35	0.63	1.58	0.86	1.19	1.07	1.03
Fe^{3+}	0.000	0.06	0.05	0.06	0.04	0.04	0.000	1.47	0.12	0.00	0.27	0.36	0.27	0.11	0.04	0.12
Mn	0.001	0.00	0.00	0.00	0.00	0.00	0.001	0.01	0.01	0.01	0.00	0.03	0.01	0.01	0.01	0.01
Mg	0.544	0.34	0.31	0.31	0.35	0.35	0.006	2.36	2.70	2.61	3.09	2.13	3.18	2.84	3.05	3.05
Ca	0.622	0.00	0.00	0.00	0.00	0.00	0.040	1.48	1.35	1.61	1.07	1.49	1.41	1.46	1.40	1.43
Na	0.331	0.10	0.10	0.10	0.09	0.09	0.970	1.05	1.01	0.88	1.12	0.95	0.81	0.87	0.86	0.89
K	0.000	0.89	0.86	0.87	0.85	0.84	0.003	0.09	0.09	0.06	0.04	0.07	0.04	0.05	0.04	0.05
Cations	3.988	7.04	6.98	6.99	6.97	6.95	5.008	15.65	15.49	15.53	15.30	15.62	15.34	15.43	15.33	15.41

Sample	YA-7-18-40														
No.	4-1	6.2	7	8.3	9	13	15	2	3	5	7.3	8.1	10	10-O	14
Mineral	Ferropargasite (inclusion)	Barroisite	Taramite (inclusion)	Magnesiohornblende	Barroisite (inclusion)	Actinolite	Barroisite	Ep (inclusion)	Ep	Ep (inclusion)	Ep	Ep	Ep	Ep	Ep
SiO_2	42.49	48.19	44.56	49.50	49.81	55.05	52.16	40.70	39.67	40.79	39.58	39.59	39.87	39.25	39.96
TiO_2	0.21	0.12	0.19	0.55	0.30	0.06	0.19	0.23	0.21	0.38	0.17	0.16	0.15	0.16	0.19
Al_2O_3	15.93	12.10	16.34	10.64	11.71	5.25	8.82	27.60	28.31	28.59	27.81	27.44	28.66	28.36	29.10
TFeO	19.42	12.31	16.49	11.03	11.21	9.17	9.24	8.35	5.86	5.78	6.67	6.97	6.00	5.99	5.98
Cr_2O_3	0.05	0.02	0.07	0.08	0.07	0.08	0.37	0.08	0.06	0.02	0.11	0.03	0.12	0.02	0.17

MnO	0.24	0.12	0.12	0.12	0.10	0.10	0.05	0.11	0.03	0.03	0.12	0.07	0.06	0.04	0.06
MgO	6.88	11.92	7.62	13.09	12.23	16.65	14.68	0.13	0.00	0.12	0.00	0.00	0.10	0.00	0.07
CaO	10.30	9.75	8.66	9.89	9.07	10.52	9.41	23.56	23.95	23.58	23.86	23.76	23.74	23.90	24.05
Na_2O	3.47	3.15	4.14	2.95	3.54	1.90	3.02	0.03	0.02	0.10	0.00	0.01	0.00	0.02	0.01
K_2O	0.37	0.36	0.50	0.29	0.35	0.28	0.26	0.01	0.00	0.05	0.04	0.01	0.00	0.00	0.00
Total	99.36	98.03	98.69	98.16	98.41	99.16	98.22	100.80	98.11	99.45	98.35	98.03	98.71	97.74	99.58
O	23.00	23.00	23.00	23.00	23.00	23.00	23.00	12.50	12.50	12.50	12.50	12.50	12.50	12.50	12.50
Si	6.31	6.93	6.52	7.06	7.07	7.65	7.34	3.127	3.048	3.134	3.041	3.041	3.063	3.015	3.070
Al	2.79	2.05	2.82	1.79	1.96	0.86	1.46	2.495	2.559	2.584	2.513	2.480	2.590	2.563	2.630
Ti	0.02	0.01	0.02	0.06	0.03	0.01	0.02	0.013	0.012	0.022	0.010	0.009	0.009	0.009	0.011
Cr	0.01	0.00	0.01	0.01	0.01	0.01	0.04	0.007	0.006	0.002	0.010	0.003	0.011	0.002	0.015
Fe^{2+}	2.08	1.30	1.84	1.23	1.32	0.89	1.09	0.000	0.000	0.000	0.000	0.000	0.000	0.000	0.000
Fe^{3+}	0.31	0.18	0.17	0.09	0.01	0.17	0.00	0.535	0.375	0.370	0.427	0.446	0.384	0.383	0.383
Mn	0.03	0.01	0.02	0.01	0.01	0.01	0.01	0.007	0.002	0.002	0.008	0.005	0.004	0.003	0.004
Mg	1.52	2.56	1.66	2.78	2.59	3.45	3.08	0.015	0.000	0.014	0.000	0.000	0.011	0.000	0.008
Ca	1.64	1.50	1.36	1.51	1.38	1.57	1.42	1.939	1.971	1.941	1.964	1.956	1.954	1.967	1.979
Na	1.00	0.88	1.17	0.81	0.97	0.51	0.82	0.005	0.003	0.015	0.000	0.001	0.000	0.003	0.001
K	0.07	0.07	0.09	0.05	0.06	0.05	0.05	0.001	0.000	0.005	0.003	0.001	0.000	0.000	0.000
Cations	15.80	15.50	15.68	15.41	15.43	15.19	15.32	8.144	7.976	8.087	7.976	7.941	8.027	7.946	8.101

Sample	YA-7-18-40					YA-7-18-47		
No.	6	8	11	8	6	5.1	5.2	6.3
Mineral	Rt	Rt	Rt	Spn	Chl (inclusion)	Rt (inclusion)	Rt	Rt
SiO_2	0.498	0.145	0.079	31.135	26.61	0.823	0.232	0.449
TiO_2	97.853	97.196	99.755	38.077	0.15	95.482	97.313	97.594
Al_2O_3	0.283	0.037	0.028	1.228	19.18	0.407	0.056	0.239
TFeO	1.013	0.253	0.233	0.358	22.12	0.995	0.317	1.345

Cr_2O_3	0.15	0.388	0.386	0.181	0.07	0.045	0.064	0.056
MnO	0.02	0.023	0	0.025	0.32	0.04	0	0.006
MgO	0.126	0.008	0.026	0.004	15.59	0.149	0.016	0.057
CaO	0.554	0.189	0.045	28.227	0.46	0.261	0.123	0.313
Na_2O	0	0	0	0.041	0.33	0.029	0.022	0
K_2O	0	0	0.008	0.011	0.18	0.01	0.004	0
Total	100.497	98.251	100.56	99.287	85.01	98.241	98.147	100.059

Notes: Superscript * means minerals used for the P–T calculation of peak metamorphism of 25–26 kbar/730–740 °C; superscript & means minerals used for the P–T calculation of retrograde metamorphism with ~7 kbar/480 ± 35 °C. Fe^{3+} of garnet is regarded as 0; Fe^{3+} estimation of omphacite is based on stoichiometry; Fe^{3+} of amphibole is based on Ref.[19]; Fe^{3+} of phengite is regard as 0.5 * total Fe. TFeO means total FeO. Spot "Line X" of garnet from YA-7-18-40 and YA-7-18-47 corresponds to garnet compositional profiles in Fig. 3a and Fig. 3b, respectively. Cations are calculated for a certain amount of oxygen listed in raw (O). The mineral abbreviations are according to Ref.[17].

Table S3. Major and trace elements.

Sample No.	YA-7-12-12	YA-7-12-13	YA-7-12-14	YA-7-18-40	YA-7-18-41	YA-7-18-42	YA-7-18-43	YA-7-18-47
XRF (wt.%)								
SiO_2	48.06	53.75	50.37	45.73	47.58	50.69	49.27	48.14
TiO_2	1.72	2.41	2.30	1.21	1.47	1.07	2.03	1.83
Al_2O_3	13.41	13.29	13.10	14.68	13.04	13.90	13.91	13.82
Fe_2O_3	14.63	14.73	16.94	11.62	14.76	11.98	15.24	14.79
MnO	0.24	0.22	0.23	0.16	0.27	0.22	0.30	0.28
MgO	6.38	4.15	6.56	9.29	7.84	7.78	6.30	6.57
CaO	11.17	5.61	6.18	11.95	10.66	9.45	7.19	8.89
Na_2O	2.53	3.41	2.60	2.01	2.22	3.22	4.00	3.01
K_2O	0.17	0.81	0.22	1.04	0.06	0.23	0.41	0.26
P_2O_5	0.13	0.33	0.20	0.14	0.17	0.09	0.21	0.20
LOI	1.93	1.29	1.45	2.38	2.34	1.50	1.36	2.87
Total	100.36	100.01	100.14	100.22	100.41	100.13	100.22	100.66
ICP–MS (ppm)								
Cr	86.13	9.86	9.90	429.67	137.75	139.09	86.77	80.11
Ni	132.56	98.62	22.06	157.30	64.75	57.95	43.37	47.40
Rb	7.27	22.89	8.99	34.95	1.94	9.30	11.82	9.14
Ba	25.38	412.78	61.72	195.40	37.93	30.66	253.39	115.84
Th	1.46	7.32	2.23	0.70	0.37	1.74	2.15	2.51
U	0.29	1.19	0.43	0.23	0.18	0.32	0.38	0.77
Nb	3.80	10.33	7.92	5.97	6.13	3.38	11.65	11.88
Ta	0.26	0.73	0.55	0.39	0.40	0.23	0.69	0.79
La	5.89	21.10	10.62	9.04	5.30	6.06	12.89	14.22
Ce	15.19	45.71	23.80	20.48	13.49	13.73	28.51	31.16
Pr	2.49	6.11	3.57	2.90	2.07	1.94	4.03	4.28
Sr	116.04	132.78	110.66	45.43	129.26	182.69	80.68	97.95
Nd	12.93	26.77	17.04	12.44	10.55	9.19	19.17	20.02
Zr	105.43	183.60	132.01	65.04	89.36	62.76	140.23	122.01
Hf	3.17	5.32	3.95	1.90	2.64	2.01	4.03	3.75
Sm	4.08	6.64	4.93	3.35	3.37	2.75	5.28	5.32
Eu	1.35	1.93	1.64	1.08	1.17	0.98	1.66	1.56
Ti	10327.52	14445.12	13824.07	7238.79	8832.99	6425.93	12170.06	10992.72
Gd	5.33	7.57	6.21	3.59	4.38	3.49	6.48	6.21
Tb	1.04	1.34	1.18	0.63	0.91	0.68	1.23	1.14
Dy	7.12	8.50	7.93	4.09	6.35	4.51	8.25	7.32
Y	39.02	44.22	42.18	21.91	34.62	23.94	45.25	39.73
Ho	1.62	1.78	1.77	0.88	1.42	0.98	1.84	1.60
Er	4.44	5.00	4.88	2.49	4.10	2.80	5.10	4.66
Tm	0.66	0.74	0.71	0.38	0.62	0.42	0.74	0.68
Yb	4.27	4.73	4.64	2.41	4.06	2.77	4.87	4.42
Lu	0.66	0.73	0.72	0.36	0.64	0.42	0.75	0.68
Li	4.21	6.22	13.52	16.49	7.69	3.80	4.86	14.47
Be	0.49	1.20	0.49	0.46	0.45	0.61	1.05	0.69
Sc	41.87	35.20	37.77	37.43	45.16	40.48	39.36	38.31
V	339.57	255.87	421.29	272.71	338.00	265.13	357.63	341.28
Sample No.	YA-7-12-12	YA-7-12-13	YA-7-12-14	YA-7-18-40	YA-7-18-41	YA-7-18-42	YA-7-18-43	YA-7-18-47
ICP–MS (ppm)								

Co	44.00	32.08	41.60	47.12	58.44	39.60	40.72	44.44
Cu	85.96	59.44	37.56	53.57	97.90	34.65	54.20	81.35
Zn	118.65	128.77	132.87	85.86	112.46	157.91	119.05	112.41
Ga	18.44	20.91	20.33	16.66	17.83	15.20	20.56	19.46
Cs	1.76	2.18	1.52	1.70	0.17	0.20	0.69	1.29
Tl	0.09	0.16	0.10	0.27	0.05	0.08	0.12	0.12
Pb	5.56	6.72	6.07	2.31	3.33	15.63	6.92	3.80
Bi	0.06	0.28	0.06	0.02	0.03	0.27	0.05	0.02
P	576.16	1439.14	857.54	616.25	751.41	376.06	916.10	890.33

Notes: "retro eclogite" means the retrograde metamorphic eclogite. LOI = loss on ignition. Detailed analytical process: About 50 mg of powder was dissolved in Teflon digesting vessel (high-pressure bomb) with equal mixture of sub-boiling, distilled HF and HNO_3, for 6 days at ~120 °C. A blank solution was prepared and the total procedural blank was <50 ng for all trace elements. An internal standard solution containing single element Indium was used to monitor signal drift during ICP–MS counting.

Table S4. SHRIMP zircon dating data.

Spots	Zircon characteristics	^{206}Pb c (%)	U (ppm)	Th (ppm)	^{232}Th /^{238}U	^{206}Pb* (ppm)	^{207}Pb* /^{206}Pb*	± %	^{207}Pb* /^{235}U	± %	^{206}Pb* /^{238}U	± %	t ^{206}Pb/ ^{238}U (Ma)	± 1σ
Eclogite sample YA-7-18-40														
1.1	homogeneous	3.39	14.85	0.03	0.0019	0.49	0.049	16.0	0.247	17.8	0.037	7.8	233.1	18.9
2.1	homogeneous	4.94	11.21	0.05	0.0044	0.37	0.056	22.2	0.279	22.7	0.036	4.4	229.4	10.9
3.1	homogeneous	2.02	26.68	0.06	0.0022	0.87	0.050	10.4	0.258	10.9	0.037	3.3	236.5	7.9
4.1	homogeneous	1.11	26.70	0.04	0.0015	0.87	0.059	9.3	0.307	9.9	0.037	3.3	236.9	7.9
5.1	homogeneous	*1.60*	*26.56*	*0.08*	*0.0032*	*0.82*	-	-	-	-	*0.035*	*3.3*	*224.6*	*7.5*
6.1	homogeneous	6.04	9.01	0.06	0.0069	0.32	0.046	27.2	0.246	27.6	0.038	4.7	243.4	12.6
7.1	homogeneous	1.89	17.80	0.08	0.0049	0.55	0.041	17.5	0.199	18.0	0.036	4.3	225.3	9.8
8.1	homogeneous	1.22	31.75	0.40	0.0132	0.93	0.054	9.6	0.249	10.2	0.034	3.3	213.0	7.1
9.1	homogeneous	6.09	26.34	0.11	0.0042	0.81	0.048	16.8	0.221	17.2	0.033	3.5	212.1	8.5
10.1	homogeneous	*6.21*	*17.69*	*0.26*	*0.0154*	*0.57*	-	-	-	-	*0.035*	*3.9*	*224.7*	*9.5*
11.1	homogeneous	3.65	10.16	0.03	0.0033	0.30	0.053	24.8	0.242	26.1	0.033	8.1	209.7	17.8
12.1	homogeneous	*4.56*	*14.05*	*0.11*	*0.0079*	*0.40*	-	-	-	-	*0.032*	*4.4*	*202.8*	*9.8*
13.1	homogeneous	*3.55*	*16.77*	*0.12*	*0.0077*	*0.63*	*0.058*	*14.2*	*0.338*	*14.8*	*0.042*	*4.0*	*267.1*	*11.2*
14.1	homogeneous	4.03	30.39	0.11	0.0038	0.87	0.060	16.1	0.267	16.7	0.032	4.1	203.9	9.1
15.1	homogeneous	2.04	42.30	0.21	0.0051	1.27	0.046	10.9	0.219	12.1	0.034	5.3	217.5	11.7
16.1	homogeneous	*2.19*	*26.39*	*0.05*	*0.0020*	*0.83*	-	-	-	-	*0.036*	*5.0*	*225.6*	*11.5*
17.1	homogeneous	2.02	23.21	0.09	0.0042	0.72	0.053	12.4	0.260	13.0	0.035	3.9	223.4	8.8
18.1	homogeneous	*23.57*	*1.89*	*0.02*	*0.0101*	*0.06*	*0.023*	*236.1*	*0.092*	*236.3*	*0.029*	*9.4*	*183.4*	*35.2*
18.2	homogeneous	*17.14*	*2.59*	*0.02*	*0.0082*	*0.07*	*-0.047*	*73.0*	*-0.165*	*71.5*	*0.025*	*8.7*	*162.0*	*23.0*
19.1	homogeneous	2.23	14.41	0.05	0.0035	0.47	0.043	18.3	0.219	18.8	0.037	4.1	233.1	9.8
20.1	homogeneous	4.38	9.55	0.04	0.0041	0.29	0.067	18.2	0.310	18.8	0.034	4.8	214.0	11.1
21.1	homogeneous	3.25	17.20	0.04	0.0025	0.55	0.051	14.8	0.253	15.3	0.036	3.8	228.7	9.2
Eclogite sample YA-7-18-47														
1.1	homogeneous	0.485	231	1	0.006	7.556	0.048	3.9	0.250	4.9	0.038	3.0	239.4	7.1
2.1	homogeneous	10.840	32	1	0.049	1.116	0.065	44.8	0.327	45.3	0.037	6.5	232.0	14.8
3.1	*core*	*2.986*	*461*	*202*	*0.452*	*23.141*	*0.062*	*16.6*	*0.484*	*16.9*	*0.057*	*3.0*	*355.1*	*10.4*
4.1	*rim*	*0.000*	*169*	*6*	*0.034*	*5.873*	*0.049*	*4.6*	*0.277*	*5.6*	*0.041*	*3.2*	*256.1*	*8.0*
5.1	*core*	*0.114*	*759*	*768*	*1.046*	*52.065*	*0.056*	*1.7*	*0.615*	*3.4*	*0.080*	*2.9*	*494.6*	*13.9*
6.1	homogeneous	2.092	22	0.2	0.010	0.697	0.055	24.6	0.273	25.0	0.036	4.4	229.5	9.9
7.1	homogeneous	3.436	32	0.1	0.003	0.936	0.056	30.7	0.252	31.0	0.032	4.2	205.8	8.5
8.1	*core*	*0.126*	*550*	*318*	*0.597*	*30.987*	*0.057*	*2.0*	*0.516*	*3.6*	*0.065*	*3.0*	*408.8*	*11.8*
9.1	*core*	*0.155*	*781*	*327*	*0.042*	*58.061*	*0.067*	*1.5*	*0.798*	*3.3*	*0.086*	*3.0*	*534.5*	*15.2*
10.1	homogeneous	0.834	100	1	0.012	3.132	0.055	26.4	0.276	26.7	0.036	4.0	229.9	9.0
11.1	homogeneous	15.003	20	0.1	0.006	0.669	0.060	47.1	0.269	47.5	0.033	6.2	206.6	12.7
12.1	rim	7.619	71	0.7	0.010	2.204	0.071	30.1	0.329	30.4	0.033	4.3	212.2	9.0
13.1	homogeneous	8.545	27	0.3	0.011	0.869	0.048	40.5	0.229	40.8	0.035	5.0	219.7	10.7
14.1	*core*	*0.000*	*3*	*0.1*	*0.024*	*0.174*	*0.373*	*10.6*	*3.465*	*17.6*	*0.067*	*14.0*	*420.1*	*57.0*

Notes: Pb c and Pb* indicate the common and radiogenic portions, respectively. Error in standard calibration was 0.30% (not included in the above errors but required when comparing data from different mounts). The spots in italic are not involved in the weighed mean ^{206}Pb/^{238}U age calculation; for detailed explanation, see the Method section. The “-” means undetected or imponderable. The results of U−Pb zircon analyses are presented as concordia plots with ellipses representing 2σ error using Wetherill concordance diagram Isoplot v.3.0[53]. Both Concordia and weighted mean ages are given in Fig. 5.
